# Simple Amides and Amines for the Synergistic Recovery of Rhodium from Hydrochloric Acid by Solvent Extraction

**DOI:** 10.1002/chem.202100630

**Published:** 2021-05-24

**Authors:** Andrew I. Carrick, Euan D. Doidge, Alexander Bouch, Gary S. Nichol, Jane Patrick, Emma R. Schofield, Carole A. Morrison, Jason B. Love

**Affiliations:** ^1^ EaStCHEM School of Chemistry University of Edinburgh Edinburgh EH9 3FJ UK; ^2^ Johnson Matthey Technology Centre Sonning Common Reading RG4 9NH UK

**Keywords:** critical metals, hydrometallurgy, recycling, sustainability, synergistic

## Abstract

The separation and isolation of many of the platinum group metals (PGMs) is currently achieved commercially using solvent extraction processes. The extraction of rhodium is problematic however, as a variety of complexes of the form [RhCl_n_(H_2_O)_6‐n_]^(n−3)−^ are found in hydrochloric acid, making it difficult to design a reagent that can extract all the rhodium. In this work, the synergistic combination of a primary amine (2‐ethylhexylamine, L^A^) with a primary amide (3,5,5‐trimethylhexanamide, L^1^) is shown to extract over 85 % of rhodium from 4 M hydrochloric acid. Two rhodium complexes are shown to reside in the organic phase, the ion‐pair [HL^A^]_3_[RhCl_6_] and the amide complex [HL^A^]_2_[RhCl_5_(L^1^)]; in the latter complex, the amide is tautomerized to its enol form and coordinated to the rhodium centre through the nitrogen atom. This insight highlights the need for ligands that target specific metal complexes in the aqueous phase and provides an efficient synergistic solution for the solvent extraction of rhodium.

## Graphical abstract

**A synergistic combination** of a simple primary amine (L^A^) and a simple primary amide (L^1^) is shown to extract rhodium from hydrochloric acid. A range of experimental and computational techniques are used to elucidate the structures of two different complexes in the organic phase; the ion‐pair [HL^A^]_3_[RhCl_6_] and the amide complex [HL^A^]_2_[RhCl_5_(L^1^)]. In the latter complex, the amide is tautomerized to its enol form and coordinated through the nitrogen atom.

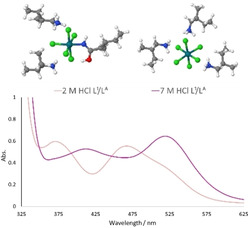

## Introduction

Rhodium is a rare platinum group metal (PGM) which is primarily used in catalytic converters to chemically reduce harmful nitrous oxide emissions to nitrogen,^[1]^ along with other uses in chemical catalysis, electronics, and jewellery. PGMs found in virgin ores or secondary sources must be separated and purified, and this is frequently achieved using hydrometallurgical methods after pyrometallurgical concentration. The hydrometallurgical process typically involves the oxidative leaching of the PGMs into hydrochloric acid and sequentially separating the metals through solvent extraction (SX), distillation of tetroxides, or precipitation methods. Currently, there is no commercial SX process for the separation of rhodium, meaning that other PGMs are generally isolated first and the rhodium subsequently recovered by precipitation with single‐use reagents.^[2]^ This process, combined with scarcity, makes rhodium the most expensive of the PGMs,^[3]^ with the highest global warming potential of any metal used in modern products.^[4]^

A major reason why the separation of rhodium by solvent extraction is challenging is its variable speciation in hydrochloric acid. A mixture of chlorido‐ and (aquo)chlorido complexes of the form [RhCl_n_(H_2_O)_6–n_]^(n−3)−^ exist in aqueous solution, depending on the age of the solution, the relative rhodium and chloride concentrations, the pH, and the temperature.^[5]^ Due to the wide range of factors affecting the speciation, there is some ambiguity in the literature with regards to the relative abundance of the complexes present.^[6]^ However, it is widely accepted that at high chloride concentrations the hexachloridometalate [RhCl_6_]^3−^ dominates, with increasing amounts of aquated complexes such as [RhCl_5_(H_2_O)]^2−^ or [RhCl_4_(H_2_O)_2_]^−^ evident as the chloride concentration diminishes. The presence of several complexes in solution makes it more difficult to achieve quantitative rhodium transport in solvent extraction processes as there is not a clear target complex to consider when designing the extractant.

There have been many attempts to develop a viable solvent extraction method for rhodium using amines,^[7]^ phosphonium ionic liquids,[^6e^, ^6f^, ^8^] sulfoxides,^[9]^ hydroxyquinolines^[10]^ and also synergistic mixtures of these extractants.^[11]^ In these cases, however, it has proven difficult to obtain selectivity over other precious metals and to extract a high percentage of rhodium without adding a large amount of tin to the feed solution; this latter process results in the formation of a Rh(I)‐Sn complex which is more readily extractable.[^7d^, ^10^] The most promising rhodium extractants to date are polydentate amidoamines which have been shown to be capable of extracting up to 90 % of rhodium from hydrochloric acid without the use of tin.[^6g^, ^12^] A key feature of these extractants is the ability to form proton chelates in which the protonated amine is stabilised by the amide carbonyl groups.[^6g^, ^12b^] This results in the formation of a charge‐diffuse array of polarised C−H^δ+^ groups which preferentially associate with the less charge‐dense (softer) chloridometalate [RhCl_5_(H_2_O)]^2−^ over the more charge‐dense (harder) chloride anions. Other amidoamine extractants have also been reported to extract [RhCl_5_(H_2_O)]^2−^ by an ion‐pair mechanism,[^12d^, ^12e^] whereas some adopt a mixed mode of action. For example, bis(acylated)diethylenetriamine extracted [RhCl_5_(H_2_O)]^2−^ by an ion‐pair mechanism over the first 10 minutes of contact, after which the formation of the inner‐sphere complex [RhCl_4_(H_2_O)(L)]^−^ dominated.^[12c]^ DFT calculations and NMR analysis suggested that the amine nitrogen atoms acted as the protonation site in the ion‐pair mechanism as well as being the donor atom for inner‐sphere binding. Tris(acylated)pentaethylenehexamine trihydrochloride was also found to extract rhodium as two complexes, the [Rh_2_Cl_9_]^3−^ metalate by an ion‐pair mechanism after short contact times, and as dinuclear complexes with either one or two chloride ligands replaced by amine nitrogen atoms after longer extraction times.^[12f]^ Despite the ability of amidoamines to extract rhodium, they are not selective over other PGMs, and highly concentrated acid or other additives such as thioureas are required to strip the loaded metals into a fresh aqueous phase.[^12b^, ^12e^] The extractants also tend to be large molecules, requiring multi‐step syntheses.

**Figure 1:**
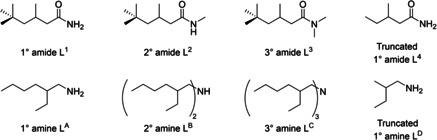
Structures of the simple amides and amines used in experimental and computational studies.

In previous work, we showed that under acidic conditions certain amidoamines decomposed into their separate ammonium and amide components yet retained their ability to transport metalates such as [ReO_4_]^−^ into an organic phase.^[13]^ This led to the discovery that the simple primary amide L^1^ (Figure 1) acted as a highly selective extractant for gold, forming supramolecular cluster assemblies in the organic phase from the initially simple components.^[14]^ It was therefore evident that a mixture of simple amide and amine compounds, instead of more sophisticated amidoamines, may facilitate the extraction of rhodium through supramolecular assembly without the need for a costly, high molecular weight extractant. The use of a synergistic mixture may also increase the likelihood of extraction by a mixed mechanism, with the different components able to extract different rhodium complexes, thereby increasing the overall amount of rhodium recovered. In this work, we evaluate combinations of simple primary, secondary, and tertiary amides, prepared by single‐step syntheses, and commercially available primary, secondary, and tertiary amines (Figure 1) as synergistic extractants for rhodium. The combination of primary amide L^1^ and amine L^A^ is found to be optimal, and the mode of extraction is determined using an array of analytical, spectroscopic, and computational analysis.

## Results and Discussion

### Solvent extraction by combinations of amides and amines

Equimolar combinations of the primary, secondary, and tertiary amides (L^1−3^) and amines (L^A−C^) were screened in spot tests for transport of rhodium into a toluene organic phase from 6 M HCl (Table 1). From these initial experiments the combination of primary amide L^1^ and primary amine L^A^ results in significantly greater extraction compared with combinations of the other amides and amines, or the use of any single reagent. The primary amine L^A^ appears to be the most important component as it is the only reagent to extract as a single component, and its performance is also boosted when combined with L^2−3^. In contrast, no extraction occurs when only the amides L^1−3^ are used. It is also interesting that little or no extraction is seen using the secondary and tertiary amines L^B^ and L^C^, respectively, given that previous attempts to extract rhodium using tertiary amines have been moderately successful.[^7b^, ^7c^, ^7d^] It should also be noted that reducing the extractant concentration greatly reduces rhodium extraction; when 0.05 M of L^1^ and 0.05 M L^A^ are used together, only 9.3 % of rhodium is extracted. At these lower initial extractant concentrations, loss of protonated extractant to the aqueous phase occurs (see later) and so there is no longer an excess of amine over rhodium to drive significant phase transport.

**Table 1:** Percentage extraction of Rh into toluene solutions by combinations of amides L^1−3^ and amines L^A−C^.^[a]^

Rh	No amide	L^1^	L^2^	L^3^
No amine	0.0	0.0	0.0	0.0
L^A^	22.5	67.2	30.4	43.8
L^B^	0.0	7.7	0.0	0.0
L^C^	0.0	0.4	0.0	0.0

[a] Conditions: Rh (0.01 M) in HCl (6 M, 2 mL) aged for 1 day prior to extraction, L^1^/L^2^/L^3^ (0.1 M) and L^A^/L^B^/L^C^ (0.1 M) in toluene (2 mL), stirred for 1 h at RT. The text colour represents the relative amount of extraction (green=100 %, gradient through to red=0 %).

A similar pattern is seen for iridium (Table 2), with maximum extraction arising from the combination of primary amines and amides, L^A^ and L^1^, respectively. The extraction of Ir(III), presumably as its hexachloridometalate [IrCl_6_]^3−^, is less common as it is typically oxidised to the Ir(IV) complex [IrCl_6_]^2−^, which is more readily extractable due to its lower aqueous hydration enthalpy.

**Table 2:** Percentage extraction of Ir into toluene solutions by combinations of amides L^1−3^ and amines L^A−C^.^[a]^

Ir	No amide	L^1^	L^2^	L^3^
No amine	0.0	0.3	0.0	0.0
L^A^	25.6	62.8	34.8	32.5
L^B^	0.0	3.1	0.1	0.0
L^C^	0.0	1.1	0.0	0.0

[a] Conditions: Ir (0.01 M) in HCl (6 M, 2 mL) aged for 1 day prior to extraction, L^1^/L^2^/L^3^ (0.1 M) and L^A^/L^B^/L^C^ (0.1 M) in toluene (2 mL), stirred for 1 h at RT. The text colour represents the relative amount of extraction (green=100 %, gradient through to red=0 %).

The selectivity of the combination of L^1^ and L^A^ for other metals was studied across a range of concentrations of HCl (Figure S1). Iridium, palladium and zinc are extracted less effectively than rhodium at almost all concentrations of HCl, 95 % of platinum is precipitated from solution by the mixture of L^1^ and L^A^, and other metals tested (Cr, Co, Ni, and Cu) are not extracted at all. This latter aspect is likely due to the difficulty in forming the chloridometalates of these metals, which requires high HCl concentrations at which competition for extraction of chloride arises. Other metals such as gold^[14]^ and tantalum^[15]^ are also extracted from HCl solutions by amide L^1^ alone. Thus overall, the combination of L^1^ and L^A^ is not particularly selective for rhodium.

**Figure 2:**
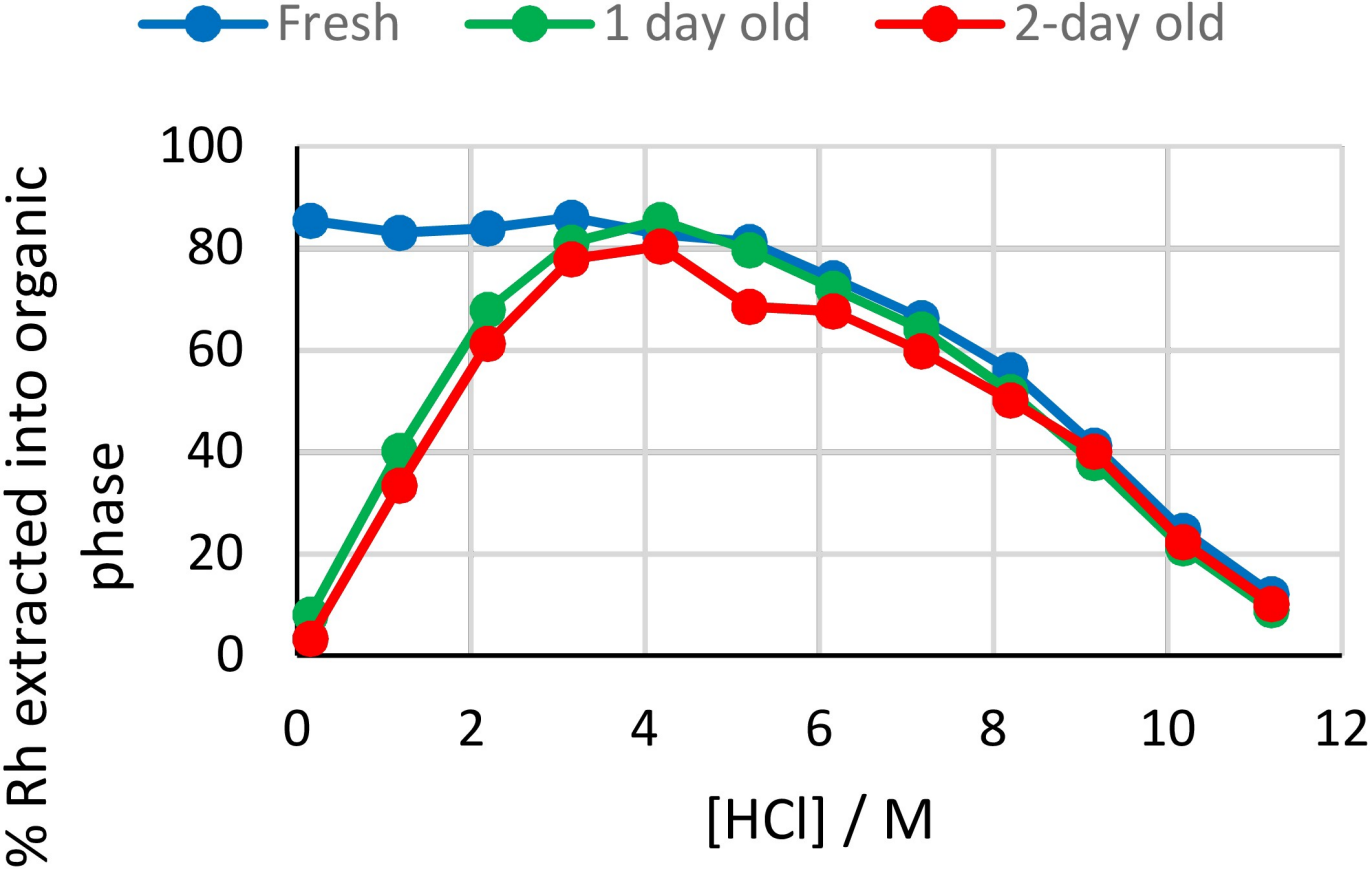
Extraction of Rh from aqueous solutions of varying age into a toluene solution containing both extractants L^1^ and L^A^. Conditions: Rh (0.01 M) in HCl (0–11 M, 2 mL) used immediately or aged for 1 or 2 days, L^1^ (0.1 M) and L^A^ (0.1 M) in toluene (2 mL), stirred for 1 h at RT.

Solvent extraction experiments using rhodium feed solutions of varying concentrations of HCl show a difference in profile for fresh and aged rhodium solutions between 0–12 M HCl. (Figure 2). Using fresh [RhCl_6_]^3−^ solutions results in a consistent extraction of approximately 85 % up to 4 M HCl before decreasing at higher [HCl] due to competition with chloride. In contrast, feed solutions which have been aged for 1 or 2 days before contact with the extractant solution show an increase from <10 % extraction at 0.15 M HCl to a peak of around 85 % at 4 M HCl before decreasing at higher [HCl]. This difference between extraction of fresh or aged samples at [HCl]<4 M indicates that aquation occurs over time at low [HCl] to produce complexes which are more difficult to extract.

The percentage of rhodium extracted into the organic phase from a fresh solution was compared with the percentage (of the original feed solution) back extracted (stripped) into ultra‐pure water (Figure S2) and shows that only a small proportion of the rhodium extracted from low [HCl] is stripped. Although less rhodium is extracted from high [HCl], almost all the extracted metal is stripped into water. This indicates that different rhodium complexes are being extracted into the organic phase at different [HCl], one of which is more readily stripped by water.

**Figure 3:**
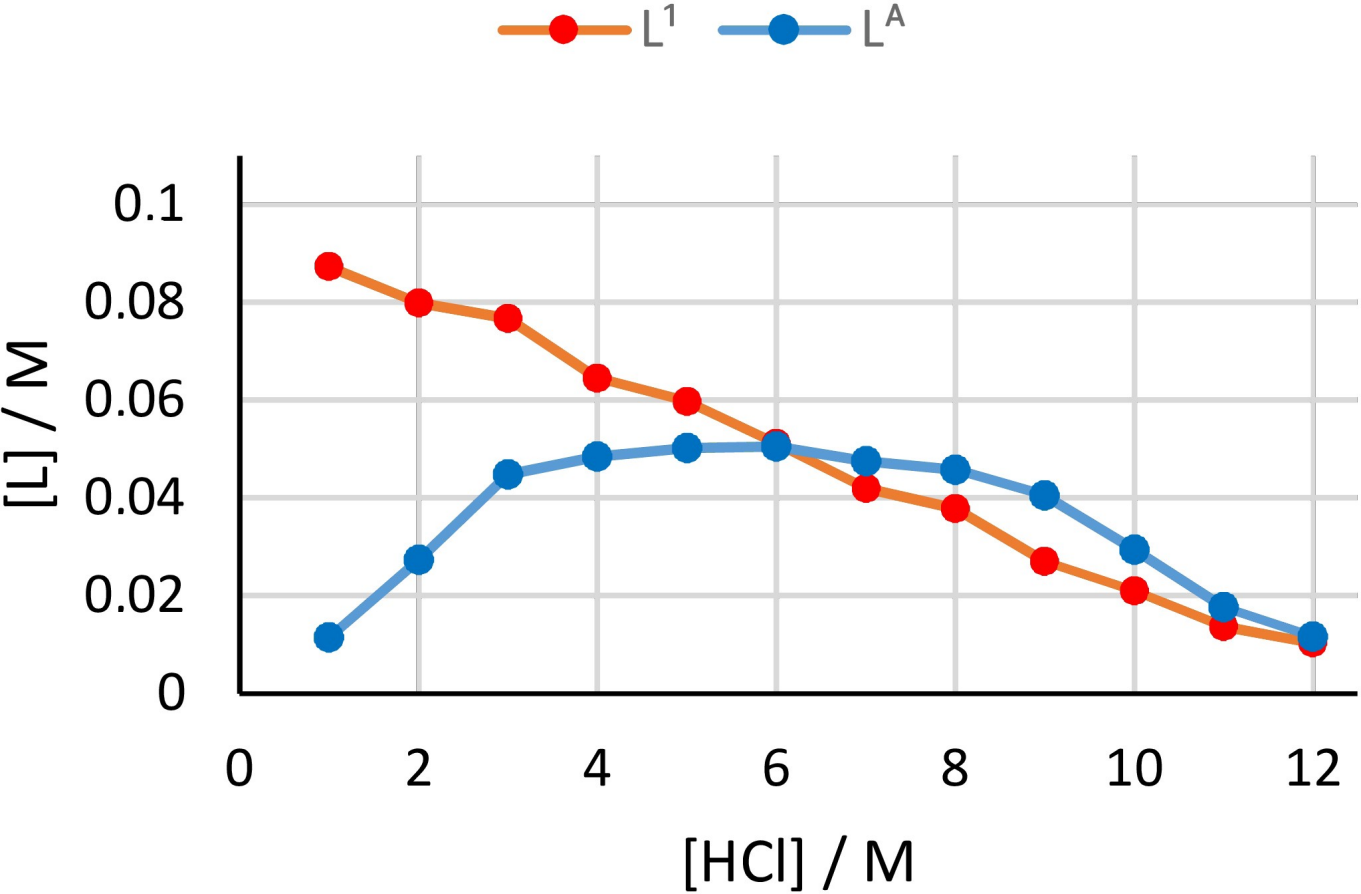
Concentrations of amide L^1^ and amine L^A^ in the organic phase (C_6_D_6_) determined by ^1^H NMR spectroscopy after extraction of Rh from solutions of varying [HCl]. Conditions: Rh (0.01 M) in HCl (1–12 M, 2 mL) aged for 1 day, L^1^ (0.1 M) and L^A^ (0.1 M) in C_6_D_6_ (2 mL), stirred for 1 h at RT.

The organic phase solubilities of L^A^ and L^1^ were analysed by quantitative ^1^H NMR spectroscopy from which it is seen that amine L^A^ has no solubility in the organic phase upon contact with aqueous HCl, i. e., all amine is protonated and resides in the aqueous phase (Figure S3). In contrast, the solubility of amide L^1^ in the organic phase is seen to decrease as the concentration of HCl increases. These trends are seen both when the extractants are alone (Figure S3) or together in the original solution (Figure S4). As L^A^ is lost to the aqueous phase with or without the presence of L^1^, this demonstrates that the synergistic effect is not simply due to enhanced organic phase solubility when the amide is present. When rhodium is present in the aqueous phase, the concentration of amine L^A^ in the organic phase increases significantly; protonated L^A^ is required to form a neutral ion pair with the rhodium chloridometalate. In contrast, the concentration of amide L^1^ in the organic phase is largely unaltered, increasing only slightly in the presence of rhodium (Figure 3).

### Identifying the mechanism of rhodium extraction

Given that the organic phase solubility of amine L^A^ is significantly enhanced in the presence of rhodium, a reverse‐micelle mechanism for rhodium extraction was initially considered.^[16]^ The water content in the synergistic organic phases after contact with aqueous acid or an acidic rhodium solution was determined by Karl‐Fischer titrations, and shows that in the absence of rhodium more water is transported into the organic phase (Figure S5). This is also the case when the concentration of one extractant is held constant and the concentration of the other extractant is increased (Figures S6 and S7), with a steeper increase in the water content in the absence of rhodium. These results therefore rule out a reverse micelle mechanism, as it would be expected that significantly more water would be transported with rhodium than without.

**Figure 4:**
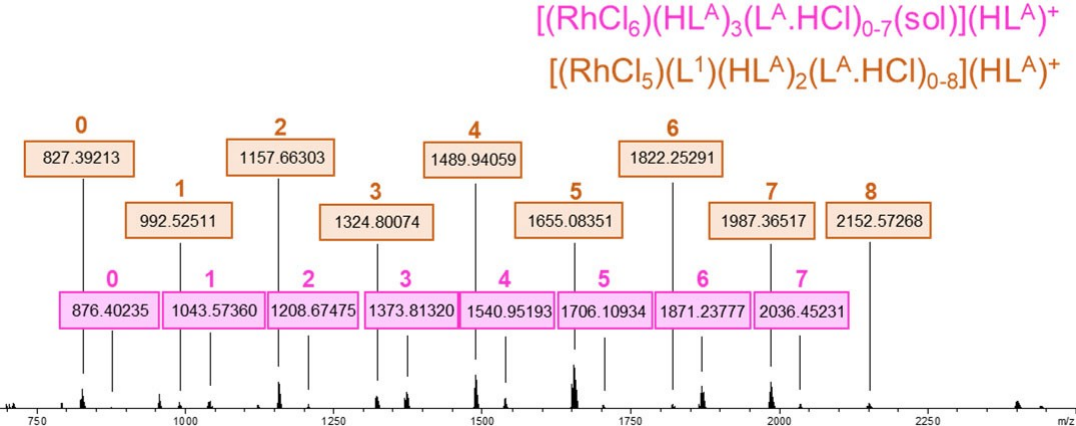
Positive‐ion ESI‐MS of a rhodium‐loaded L^1^/L^A^ toluene solution diluted in CH_3_CN. sol=CH_3_CN. Assignments of peaks are noted in Figures S8 and S9.

Positive‐ion ESI‐MS experiments were undertaken to gain further insight into the identity of the rhodium complex(es) extracted into the organic phase following contact with a 1‐day‐aged 0.01 M rhodium in 4 M HCl solution. These data show that two sets of rhodium‐containing ions are present (Figure 4), one with the general formula [(RhCl_6_)(HL^A^)_3_(L^A^ ⋅ HCl)_0‐7_(MeCN)](HL^A^)^+^ and the other [(RhCl_5_)(L^1^)(HL^A^)_2_(L^A^ ⋅ HCl)_0‐8_](HL^A^)^+^.

Further ESI‐MS experiments recorded after very short, 5 second contacts show that only the [RhCl_6_]^3−^ set of ions are present in the organic phase, indicating that the pentachloridorhodate anion is extracted more slowly and requires the presence of amide L^1^. In contrast, the ESI‐MS of the organic phases of iridium extractions by L^1^/L^A^ mixtures show only ions containing the hexachloridometalate [IrCl_6_]^3−^ (Figure S10). The lack of [IrCl_5_]^2−^ complexes or iridium complexes containing L^1^ is consistent with the higher substitutional inertness of Ir(III) compared with Rh(III), resulting in lower concentrations of aquated complexes of iridium in the aqueous phases.

**Figure 5:**
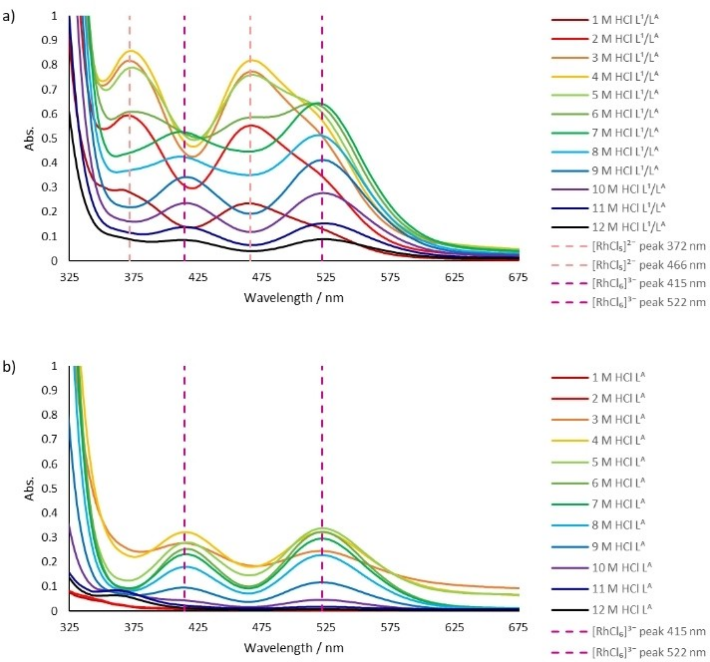
UV‐vis spectra of organic phase containing both L^1^/L^A^ extractants (a) or L^A^ only (b) after 1 h contact with solutions of Rh in varying [HCl]. Conditions: Rh (0.01 M) in HCl (1–12 M, 2 mL) aged for 1 day, L^1^ (0.1 M) and L^A^ (0.1 M) or L^A^ (0.1 M) in toluene (2 mL), stirred for 1 h at RT.

The presence of different rhodium complexes in the organic phases after extraction by L^1^/L^A^ mixtures is evident from the colours of the organic phases, which range from peach to cherry pink (Figure S11) This is evidenced in the UV‐vis spectra of the rhodium‐loaded organic phase after synergistic extraction from a range of HCl concentrations by L^A^/L^1^ mixtures (Figure 5a). These spectra show that two compounds are extracted, one of which predominates when extracting from 1–3 M HCl (peak maxima at 466 and 372 nm), with a mixture present when extracting from 4–7 M HCl, while a second compound dominates at >7 M HCl (peak maxima at 522 and 415 nm).

In contrast, the UV‐vis spectra of the organic phases after an extraction with the amine L^A^ only show that a single complex is extracted, with peak maxima at 522 and 415 nm (Figure 5b). Comparison of the peak wavelengths with those described for rhodium aqueous phases suggests that this feature is due to the hexachloridometalate [RhCl_6_]^3−^.[^6e^, ^6f^] It is therefore evident that the organic phase of the synergistic extraction (Figure 5a) comprises a mixture of [RhCl_6_]^3−^ and [RhCl_5_(L^1^)]^2−^ metalates. These conclusions agree with the ESI‐MS data which show two sets of ions for [RhCl_6_]^3−^ and [RhCl_5_(L^1^)]^2−^. Therefore, it is evident that the extraction of [RhCl_6_]^3−^ is achieved using the amine L^A^ only whereas the amide L^1^ is also required for the extraction of [RhCl_5_]^2−^ ions.

Although using the secondary or tertiary amides L^2^ or L^3^ with L^A^ results in increased extraction compared with L^A^ alone, it is interesting to note that no absorptions due to [RhCl_5_]^2−^ are observed in the UV‐vis spectra of rhodium‐loaded organic phases following extraction from 4 M HCl (Figure S12). This indicates that these amides do not coordinate to Rh, forming [RhCl_5_(L^2/3^)]^2−^, so the improved extraction is likely due to a solvating effect whereby the amide carbonyl group may displace water molecules which typically solvate primary ammonium groups.^[17]^ It also suggests that replacing the amide N−H with N−Me disfavours the formation of a [RhCl_5_(L^2^/^3^)]^2−^ complex, either as a result of steric hindrance or an inability of the amide to adopt an enol structure (see later).

The evolution of the speciation in the organic phase when disengaged from the aqueous phase was monitored by UV‐vis spectrophotometry. As seen when in the ESI‐MS spectra, the UV‐vis spectra of the disengaged organic phase after a 5 second extraction comprises almost exclusively the [RhCl_6_]^3−^ complex (peak maxima at 522 and 417 nm) (Figure S13). However, in the subsequent 24 h there are significant shifts in the UV‐vis spectra, showing the growth of absorptions due to the [RhCl_5_(L^1^)]^2−^ complex (peak maxima at 468 and 374 nm). After 24 h, the equilibrium between rhodium chloridometalate species has stabilised, with approximately the same ratio of rhodium complexes expressed as obtained after a 1‐hour synergistic extraction (Figure 5a). The rapid extraction of the [RhCl_6_]^3−^ complex suggests an outer‐sphere ion‐pair mechanism, while the longer time required for the extraction or formation of a [RhCl_5_(L^1^)]^2−^ complex is indicative of an inner‐sphere mechanism, with the amide L^1^ replacing a chloride or water ligand. Further UV‐vis analysis of the organic phase (Figure S14) after stripping into a fresh aqueous phase shows that water can readily strip the [RhCl_6_]^3−^ complex. In contrast, contact with 10 M HCl is required to totally strip the [RhCl_5_(L^1^)]^2−^ complex, most likely due to an excess of chloride ions acting to displace the inner‐sphere amide ligand and to form [RhCl_6_]^3−^, which is more easily transported into the aqueous phase.

**Figure 6:**
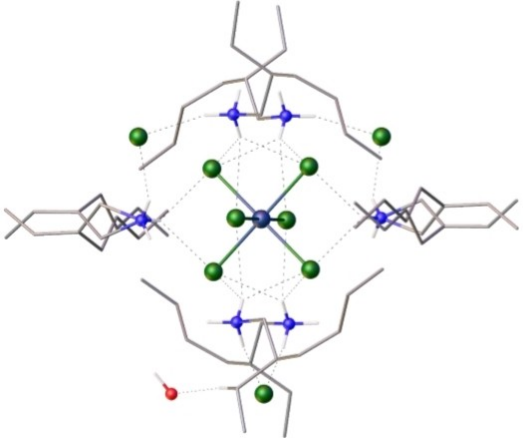
X‐ray crystal structure of [(HL^A^)_3_RhCl_6_(HL^A^Cl)_3_(H_2_O)] showing the simplest complete unit. (For clarity, all hydrogen atoms are omitted except those on nitrogen atoms; grey C, red O, blue N, white H, green Cl, purple Rh.) Deposition number 2060879 contains the supplementary crystallographic data for this paper. These data are provided free of charge by the joint Cambridge Crystallographic Data Centre and Fachinformationszentrum Karlsruhe Access Structures service

The relatively high baseline absorbance for the amine‐only (L^A^) organic phases contacted with rhodium at 3, 4 and 5 M HCl (Figure 5b) is caused by light scattering due to the formation of fine pink crystals. These crystals are observed in both duplicates for these samples, but not in the samples extracted using the L^A^/L^1^ synergistic mixture. Similar crystals develop over a period of several months following the slow evaporation of a rhodium‐loaded toluene phase from a synergistic extraction. Both sets of crystals have the same unit cell parameters, and the X‐ray crystal structure (Figure 6 and supplementary information) reveals a central, octahedral [RhCl_6_]^3−^ anion encapsulated by six protonated amines. The arrangement of the ammonium cations is reinforced through further interactions with three exogenous chloride ligands and a single molecule of water, resulting in an overall formula of [(HL^A^)_3_RhCl_6_(HL^A^Cl)_3_(H_2_O)]. The ratio of six ammonium cations and three chloride anions per [RhCl_6_]^3−^ metalate is similar to that previously seen following the precipitation of rhodium using 4‐alkylanilines,^[18]^ although this structure did not contain a water molecule. Although a solid‐state structure is not a true representation of a solvent extraction system, the crystallisation of [(HL^A^)_3_RhCl_6_(HL^A^Cl)_3_(H_2_O)] which is free from L^1^ reinforces the conclusions from the ESI‐MS and UV‐visible data described above, namely that L^A^ alone extracts [RhCl_6_]^3−^, and that the metalate is stabilised in the organic phase by outer‐sphere interactions. Moreover, as these crystals were obtained much more quickly from an extraction using only the amine L^A^ than from a synergistic extraction, this supports the suggestion that the amide helps to solubilise the [RhCl_6_]^3−^ in the organic phase through a solvation effect.

### Formation and structural analysis of [RhCl_5_(L^1^)]^2−^

**Figure 7:**
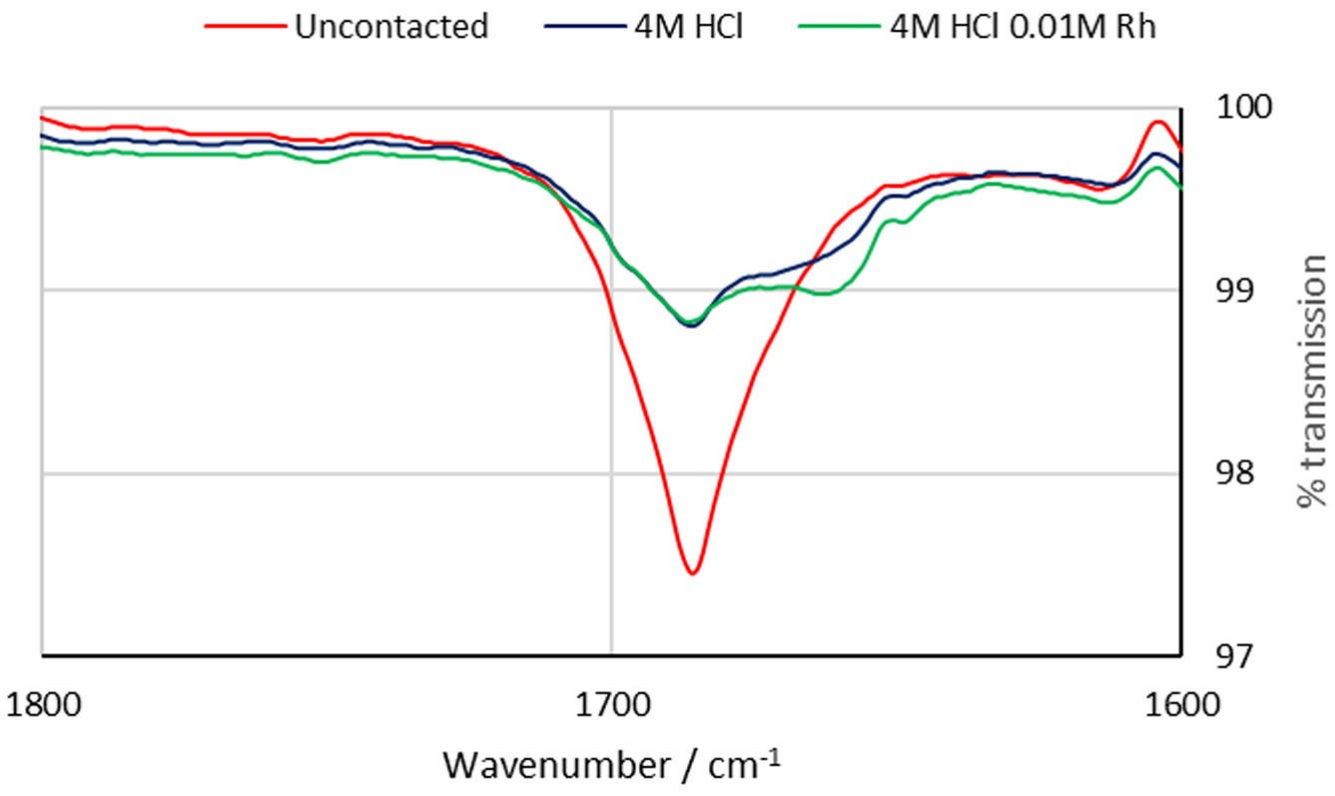
FT‐IR spectra of the organic phase showing the C=O stretching frequency. Conditions: Rh (0.01 M) in HCl (4 M, 2 mL) aged for 1 day, L^1^ (0.1 M) and L^A^ (0.1 M) in toluene, stirred for 1 h at RT.

As crystals could not be obtained that contained either [RhCl_5_]^2−^ or amide L^1^, alternative techniques were used to investigate how amide L^1^ may bind to rhodium to form the extracted complex. The FT‐IR spectrum of the organic phase after contacting the synergistic mixture with 4 M HCl shows a considerable reduction in the intensity of the C=O stretch of the amide at 1686 cm^−1^ compared with the uncontacted organic phase. This is to be expected due to the loss of the amide to the aqueous phase at this concentration of HCl (see Figure S4). After contact with a 4 M HCl solution containing 0.01 M rhodium, a small but distinct second peak is observed at 1663 cm^−1^ in the IR spectrum (Figure 7), representing a shift of 23 cm^−1^ from the uncontacted amide L^1^. This is indicative of weakening of the C=O bond but does not differentiate between coordination of the amide to rhodium centre or hydrogen bonding to a proton.

**Figure 8:**
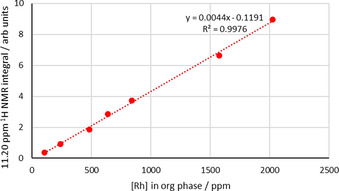
Integral of ^1^H NMR resonance at 11.21 ppm in the organic phase after extraction plotted against [Rh] in the organic phase measured by ICP‐OES. Conditions: Rh (0.001–0.050 M) in HCl (4 M, 2 mL) aged for 1 day, L^1^ (0.1 M) and L^A^ (0.1 M) in toluene or C_6_D_6_ (2 mL), stirred for 1 h at RT.

**Figure 9:**
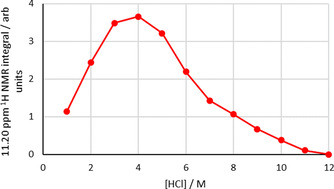
Integral of ^1^H NMR resonance at 11.21 ppm in the organic phase after extraction plotted against [HCl] in the aqueous phase. Conditions: Rh (0.01 M) in HCl (1–12 M, 2 mL) aged for 1 day, L^1^ (0.1 M) and L^A^ (0.1 M) in C_6_D_6_ (2 mL), stirred for 1 h at RT.

**Figure 10:**
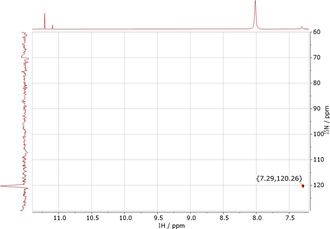
^1^H‐^15^N HSQC spectra of a Rh‐loaded organic phase. Conditions: Rh (0.05 M) in HCl (2 M, 2 mL) aged for 1 day, L^1^ (0.1 M) and L^A^ (0.1 M) in C_6_D_6_ (2 mL), stirred for 1 h at RT.

**Figure 11:**
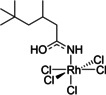
Likely structure of [RhCl_5_(L^1^)]^2−^ complex from NMR analysis (only inner‐sphere ligands are shown).

The ^1^H NMR spectrum of the organic phase (C_6_D_6_) after a 1 h synergistic extraction shows new resonances at 7.29 and 11.21 ppm. These peaks are not present following an amine‐only or amide‐only extraction and are not observed when the synergistic extractant solution is contacted with aqueous HCl solutions that do not contain rhodium. The integrals of these resonances are identical but the resonance at 11.21 ppm is more clearly resolved and so was used in quantitative analysis. Increasing the concentration of rhodium in the aqueous feed solution from 0.001 to 0.050 M causes the absolute concentration of rhodium in the organic phase to increase (Figure S14). Plotting the integral of the ^1^H NMR resonance at 11.21 ppm against the concentration of rhodium in the organic phase gives a straight‐line correlation (Figure 8), strongly suggesting that an extracted rhodium complex is responsible for this resonance in the ^1^H NMR spectrum. A plot of the integral of this peak as a function of initial [HCl] of the aqueous feed solution (Figure 9) gives a similar plot to that of % rhodium extraction from aged solutions (Figure 2), reaching its highest value at 4 M HCl. Following a D_2_O shake, both the resonances at 11.21 and 7.29 ppm disappear; as such, these protons are ionisable and are therefore likely to be bound to either oxygen or nitrogen (or both). The ^1^H‐^15^N correlation spectra (Figure 10) show a cross peak for the proton signal at 7.29 ppm, indicating this proton is bonded to nitrogen, but is absent for the resonance at 11.21 ppm, which means this proton is likely bound to oxygen. As only one proton is bound to nitrogen, this indicates that the amide L^1^ has tautomerised to its enol form and is likely coordinated to the rhodium through the nitrogen atom (Figure 11).

**Figure 12:**
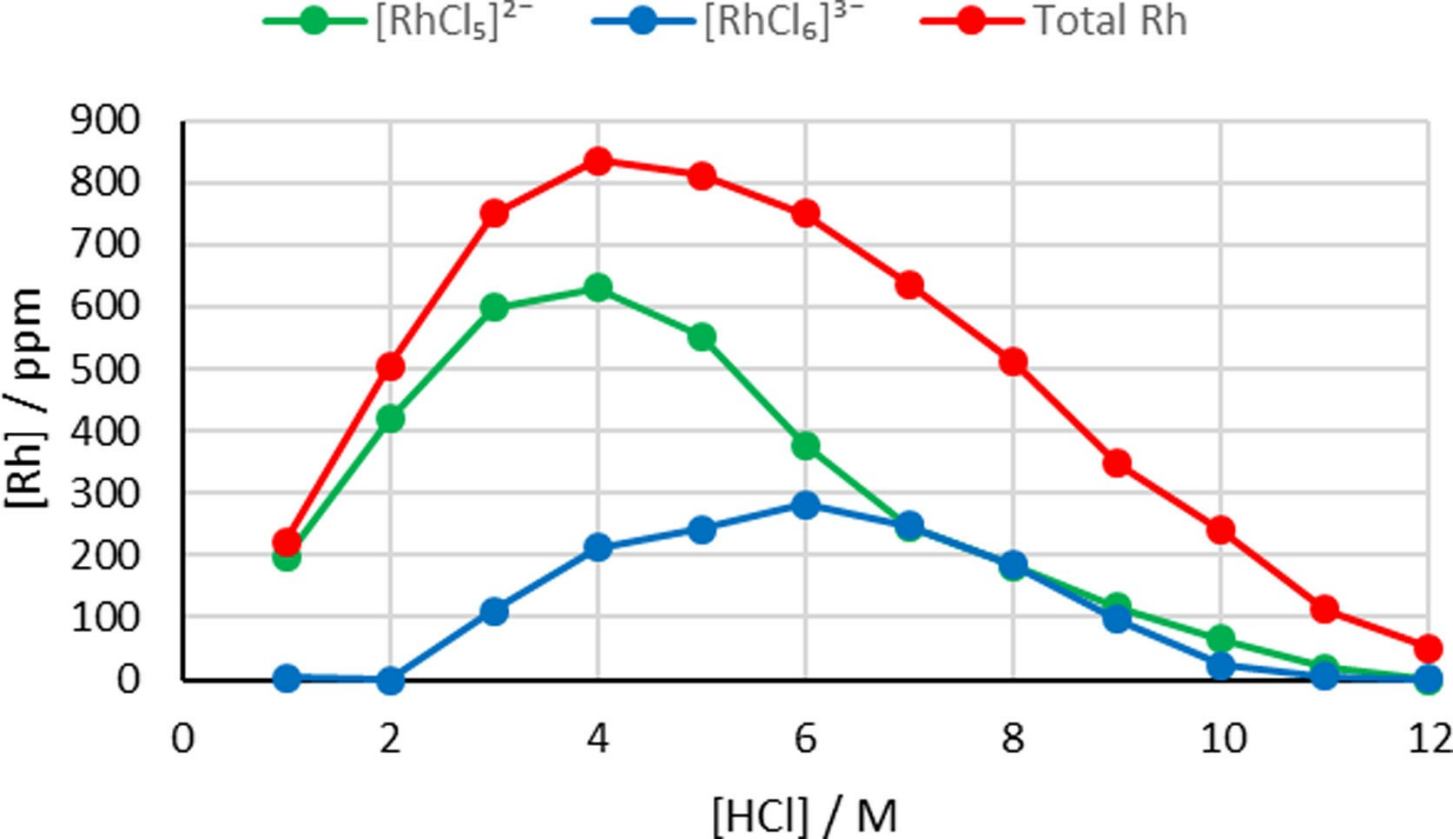
Concentration of rhodium complexes in organic phase following extraction from varying [HCl]. Concentration of total Rh and [RhCl_6_]^3−^ determined by ICP‐OES analysis following synergistic and amine‐only extractions, respectively. Possible concentration of [RhCl_5_]^2−^ determined by ^1^H NMR analysis. Conditions: Rh (0.01 M) in HCl (1–12 M, 2 mL) aged for 1 day, L^1^ (0.1 M) and L^A^ (0.1 M) or L^A^ (0.1 M) in toluene or C_6_D_6_ (2 mL), stirred for 1 h at RT.

Assuming the peak at 11.21 ppm is due to one proton and is part of the extracted [RhCl_5_(L^1^)]^2−^ complex allows the concentration of this complex to be determined from the NMR data and compared with the concentrations determined by ICP‐OES analysis for both synergistic (to determine the total concentration of rhodium) and amine‐only extractions (to determine [RhCl_6_]^3−^) (Figure 12). At 1–6 M HCl, combining the [RhCl_5_]^2−^ concentration with that for [RhCl_6_]^3−^ gives a summation very close to the total [Rh] determined by ICP‐OES. At higher [HCl], the value obtained by combining the concentrations of the two species is lower than the total [Rh] which may be due to the solubilising of [HL^A^]_3_[RhCl_6_] by the amide. Overall, this work explains the shape of the rhodium‐extraction profile vs [HCl], with the extraction of [RhCl_5_(L^1^)]^2−^ dominating at lower [HCl].

### Computational modelling

**Figure 13:**
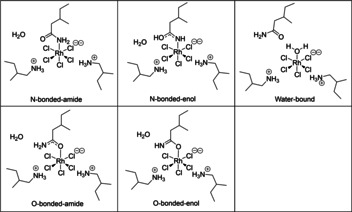
Computational models investigated to explore coordination modes for [HL^D^]_2_[RhCl_5_(L^4^)](H_2_O).

The most energetically favourable structure of [RhCl_5_(L^1^)]^2−^ was investigated using DFT calculations. To reduce the computational cost of the geometry optimisation process, calculations were carried out using the truncated versions of the amine and amide extractants, L^D^ and L^4^, respectively (Figure 1). Optimisations were carried out on at least three different starting geometries for each model to increase the probability of finding the global energy minima in each case. A range of different [RhCl_5_(L^4^)]^2−^ complexes with the amide bound to rhodium by nitrogen or oxygen atoms (in both the amide and enol tautomeric forms), as well as a complex in which the amide is hydrogen bonded to an inner‐sphere water molecule, were modelled to investigate the most favourable mode of binding. Two outer‐sphere ammonium cations, (HL^D^)^+^, were included in each model to form an overall charge‐neutral assembly. An outer‐sphere water molecule was also included in each of the models with an inner‐sphere amide L^4^ to achieve a consistent atom‐count across all models (Figure 13); as a result, their Gibbs free energy corrected optimisation energies can be compared directly.

**Figure 14:**
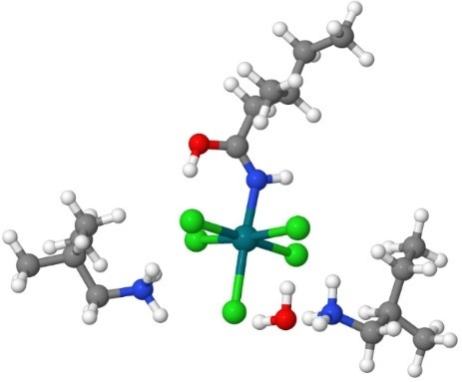
Lowest energy structure of [HL^D^]_2_[RhCl_5_(L^4^)](H_2_O) complexes showing the enol tautomer of L^4^ bound to rhodium (dark green) by the N atom (blue).

Considering the lowest energy value calculated for each model (Table 3, Column 1), complexes with the inner‐sphere amide L^4^ bound through the nitrogen atom are the most stable, with the enol‐tautomer predicted to be more stable than the amide (Figure 14). The complex with an inner‐sphere water molecule is higher in energy, while binding through the oxygen atom on amide L^4^ yields local minima that are considerably higher in energy, particularly for the enol form. These calculations agree with the experimental data, as the relatively slow extraction of [RhCl_5_(L^1^)]^2−^ is a consequence of slow inner‐sphere ligand substitution kinetics, and the multinuclear NMR analysis supports tautomerisation of the amide to its enol form. The lower energy of the enol tautomer also helps to explain why the secondary and tertiary amides L^2^ and L^3^ are less effective synergists than L^1^. It must be cautioned, however, that even with truncated R‐groups these models were found to comprise many local energy minima (Table S1), which reflects the large number of degrees of freedom that exist through ligand twisting, and the positioning of the (HL^D^)^+^ cations and the coordinated water molecule. Simplifying the model through removal of the loosely bound outer‐sphere water molecule (Table 3, Column 2), and further to remove the (HL^D^)^+^ cations (Table 3, Column 3), still supports favourable binding of the amide ligand through the nitrogen atom rather than the oxygen atom. However, in these cases, the N‐bound‐amide tautomer is now the lowest in energy, indicating that outer‐sphere interactions with the ammonium ions and coordinated water molecule must play a stabilising role in the formation of the N‐bound‐enol tautomer. Furthermore, it is evident from the crystal structure of the [RhCl_6_]^3−^ complex (Figure 6) that six ammonium cations along with three exogenous chloride ions cluster around the metalate, and so further intermolecular interactions may be present in solution to stabilise the extracted complex. While it would therefore be of interest to investigate a larger number of ammonium cations clustered around the [RhCl_5_(L^1^)]^2−^ complex, DFT calculations for these models would be computationally demanding, and the high degrees of freedom would likely present an intricate potential energy landscape with many low‐lying local energy minima.

**Table 3:** Free energy values (kJ mol^−1^) relative to the lowest energy structure for models containing [HL^D^]_2_[RhCl_5_(L^4^)](H_2_O), [HL^D^]_2_[RhCl^5^(L^4^)] and [RhCl_5_(L^4^)]^2−^ with different binding modes to the Rh centre.^[a]^

Complex	[HL^D^]_2_[RhCl_5_(L^4^)](H_2_O)	[HL^D^]_2_[RhCl_5_(L^4^)]	[RhCl_5_(L^4^)]^2−^
N‐bonded‐amide	12.2	0.0	0.0
N‐bonded‐enol	0.0	5.1	31.5
O‐bonded‐amide	44.0	25.9	48.3
O‐bonded‐enol	126.4	116.6	77.7
Water‐bound	37.2

[a] Free energy values calculated using M06/6‐31+G*. Complexes in each column are coloured relative to the lowest energy structure (lowest energy in green, highest energy in red).

## Conclusion

We have shown that a combination of the simple primary amide L^1^ and primary amine L^A^ can synergistically extract greater than 80 % of rhodium from a hydrochloric acid solution. While the system is only partially selective over other transition metals such as iridium, the level of rhodium extraction is similar to that achieved using other extraction systems which do not use tin(II) chloride and has the benefit of using much simpler extractants relative to those reported previously.

At high [HCl], or when a freshly prepared solution of [RhCl_6_]^3−^ is used, rhodium is primarily transported into the organic phase as the ion‐pair [(HL^A^)_3_(RhCl_6_)] that is straightforwardly recovered by a water strip. However, if the feed solution is aged for at least one day prior to extraction, at lower [HCl], inner‐sphere complexation of the amide L^1^ occurs with subsequent co‐transport of [(HL^A^)_2_RhCl_5_(L^1^)]; this latter complex is also formed in the organic phase over time. Computational modelling and multinuclear NMR spectroscopy concur and demonstrate that the coordination of amide L^1^ occurs by the nitrogen atom rather than the oxygen, and that the amide has tautomerised to its enol form; this feature may explain why the secondary L^2^ and tertiary L^3^ amides do not easily form inner‐sphere complexes and are hence less effective extractants of [RhCl_5_]^2−^.

The synergistic mixture used in this study maximises the extraction of rhodium by adapting to the dynamic speciation of rhodium in aqueous solution. The potential difficulty of extracting aquated chlorometalates is negated by the ability of the primary amide L^1^ to bind directly to the metal. It is perhaps surprising that the extraction of [RhCl_6_]^3−^ by the simple primary amine L^A^ occurs, given the high hydration enthalpy of this ion in the aqueous phase. It is also evident that its extraction is enhanced by the presence of amide L^1^, which is likely due to the amide displacing waters of hydration which would otherwise associate with the ammonium cations. Enhanced extraction in the presence of amide L^1^ is also seen for [IrCl_6_]^3−^ which is kinetically inert and for which no inner‐sphere binding of L^1^ is observed. While the synergistic mixture is not particularly selective for rhodium over other PGMs, the relative simplicity of the compounds L^1^ and L^A^ is a significant advantage over more complex molecules which achieve similar levels of extraction.

## Conflict of interest

The authors declare no conflict of interest.

## Supporting information

As a service to our authors and readers, this journal provides supporting information supplied by the authors. Such materials are peer reviewed and may be re‐organized for online delivery, but are not copy‐edited or typeset. Technical support issues arising from supporting information (other than missing files) should be addressed to the authors.

Supplementary
