# Supplementary material for: Simple Amides and Amines for the Synergistic Recovery of Rhodium from Hydrochloric Acid by Solvent Extraction
Source: Chemistry. 2021 May 24;27(34):8714–22. doi: 10.1002/chem.202100630 (PMC8252629; doi:10.1002/chem.202100630)
Supplement: Supplementary file 1 — Supplementary [file CHEM-27-8714-s001.pdf]

Chemistry–A European Journal

Supporting Information

Simple Amides and Amines for the Synergistic Recovery of Rhodium from Hydrochloric Acid by Solvent Extraction

Andrew I. Carrick, Euan D. Doidge, Alexander Bouch, Gary S. Nichol, Jane Patrick, Emma R. Schofield, Carole A. Morrison,* and Jason B. Love*

Table of Contents

Experimental **S2**
Extraction of Different Metals **S4**
Stripping Data **S4**
Extractant Solubility Plots **S5**
KF Titration Data **S6**
ESI-MS Spectra **S8**
Organic Phase Speciation **S10**
Crystallography of $[RhCl_6]^{3-}$ complex **S12**
Extraction of Rhodium from Varying Feed Concentrations **S13**
Acid-Base Titration **S14**
Computational Data **S15**

Experimental

Reagents and Synthesis

All solvents and reagents were used as received from Acros Organics, Alfa Aesar, Fisher Scientific, Johnson Matthey, Merck Millipore, Sigma-Aldrich or VWR International. Deionised water was sourced from a Milli-Q reverse osmosis system. The amides L^1, L^2 and L^3 were synthesised using previously reported methods.[1]

Solvent Extractions

An aqueous phase containing Na_3RhCl_6 (0.01 M) in varying concentrations of aqueous HCl (0 - 12 M, 2 mL), freshly prepared or aged for 1 or 2 days at RT, was contacted with an organic phase containing one or both extractants (0.1 M) in toluene (2 mL) for 1 h at RT with magnetic stirring at 750 rpm. After mixing and phase disengagement, the phases were separated and analysed for metal content by ICP-OES.

Stripping of Loaded Organic Phase

The post-extraction organic phase (1.5 mL) was contacted with an aqueous stripping solution (1.5 mL) for 1 h at RT with magnetic stirring. After mixing and phase disengagement, the phases were separated and analysed for metal content by ICP-OES.

ICP-OES

Following phase separation, an yttrium internal standard (1000 ppm, 0.1 mL) and 1-methoxy-2-propanol (9.8 mL) were added to the sample (0.1 mL). Analyses were repeated in duplicate for both the organic and aqueous phases using either a Perkin Elmer Optima 8300 or a 5300DC Inductively Coupled Plasma Optical Emission Spectrometer.

ESI-MS

ESI FT-ICR MS measurements were recorded in positive-ion mode using the standard Bruker ESI sprayer operated in “infusion” mode coupled to a SolariX FTICR mass spectrometer. Direct infusion spectra were typically a sum of 20 acquisitions. All mass spectra were analysed using DataAnalysis software version 4.4 (Bruker Daltonics) with the ions assigned manually.

UV-visible Spectrophotometry

The neat organic phase solutions from solvent extraction experiments were analysed against a solvent blank over the range 300 – 900 nm on a Shimadzu UV-1900 spectrometer.

X-ray Crystallography

Single light-pink plate-shaped crystals of $[(HL^A)_3RhCl_6(L^A.HCl)_3(H_2O)]$ crystallised from a rhodium-loaded organic phase by slow evaporation. A suitable crystal (0.53×0.45×0.03) mm^3 was selected and mounted on a MITIGEN holder in Paratone oil on a Rigaku Oxford Diffraction SuperNova diffractometer. The crystal was kept at T = 120.0 K during data collection. The structure was solved by direct methods using ShelXT[2] within Olex2,[3] and was refined by least squares minimisation using version 2014/7 of ShelXL.[2]

FT-IR Spectroscopy

The neat organic phase solutions from solvent extraction experiments were analysed against a solvent blank over the range 1400 – 2400 cm^{-1} on a Perkin Elmer Spectrum 65 FT-IR spectrometer.

NMR Spectroscopy

Extractions were performed using C_6D_6 as the organic phase instead of toluene. For quantitative analysis, dioxane (1 M) in C_6D_6 (0.025 mL) was added to the organic phase (1 mL) following phase separation. ^1H NMR spectra were recorded on Bruker AVA500 or AVA600 spectrometers at 300 K at 500 or 600 MHz. ^1H-^{15}N HSQC spectra were recorded on a Bruker AVA800 spectrometer at 298 K at 800 MHz for ^1H and 81.0 MHz for ^{15}N. Spectra were referenced internally to residual protio solvent, and chemical shifts are reported in δ (ppm).

Karl Fischer Titrations

Organic phase samples were analysed in triplicate on a Mettler Toledo C30S Coulometric KF titrator using a DM 143-SC electrode using HYDRANAL Coulomat AD as the titration reagent.

Acid-Base Titrations

Organic phase samples (1 mL) were diluted in propan-2-ol (4 or 8 mL) and a few drops of phenolphthalein indicator was added. The samples were manually titrated against NaOH (0.005 or 0.01 M) until a colour change from colourless to pink was observed.

Computational Modelling

DFT geometry optimisation calculations were carried out using Gaussian16,[4] at the M06 level of theory.[5] A LANL2DZ pseudopotential basis set[6] was used for rhodium, and the 6-31+G* basis set[7] for all other atoms. Polarization Continuum Models (solvent = water) were used to implicitly mimic the solvation of structures in the aqueous phase. The truncated versions of the extractants L^D and L^4 were used to reduce the computational demand of the calculations. Structures were optimised to standard convergence criteria and confirmed as minima through subsequent vibrational frequency calculation (all eigenvectors real numbers). The vibrational frequency calculations also provided the required thermodynamic corrections to recast the optimisation energies as free energy values.

References

[1] a) E. D. Doidge, I. Carson, P. A. Tasker, R. J. Ellis, C. A. Morrison, J. B. Love, *Angew. Chem. Int. Ed.* **2016**, *55*, 12436-12439; b) E. D. Doidge, L. M. M. Kinsman, Y. Ji, I. Carson, A. J. Duffy, I. A. Kordas, E. Shao, P. A. Tasker, B. T. Ngwenya, C. A. Morrison, J. B. Love, *ACS Sustainable Chem. Eng.* **2019**, *7*, 15019-15029.

[2] G. Sheldrick, *Acta Crystallographica Section A* **2015**, *71*, 3-8.

[3] O. V. Dolomanov, L. J. Bourhis, R. J. Gildea, J. A. K. Howard, H. Puschmann, *J. Appl. Crystallogr.* **2009**, *42*, 339-341.

[4] M. J. Frisch, G. W. Trucks, H. B. Schlegel, G. E. Scuseria, M. A. Robb, J. R. Cheeseman, G. Scalmani, V. Barone, G. A. Petersson, H. Nakatsuji, X. Li, M. Caricato, A. V. Marenich, J. Bloino, B. G. Janesko, R. Gomperts, B. Mennucci, H. P. Hratchian, J. V. Ortiz, A. F. Izmaylov, J. L. Sonnenberg, Williams, F. Ding, F. Lipparini, F. Egidi, J. Goings, B. Peng, A. Petrone, T. Henderson, D. Ranasinghe, V. G. Zakrzewski, J. Gao, N. Rega, G. Zheng, W. Liang, M. Hada, M. Ehara, K. Toyota, R. Fukuda, J. Hasegawa, M. Ishida, T. Nakajima, Y. Honda, O. Kitao, H. Nakai, T. Vreven, K. Throssell, J. A. Montgomery Jr., J. E. Peralta, F. Ogliaro, M. J. Bearpark, J. J. Heyd, E. N. Brothers, K. N. Kudin, V. N. Staroverov, T. A. Keith, R. Kobayashi, J. Normand, K. Raghavachari, A. P. Rendell, J. C. Burant, S. S. Iyengar, J. Tomasi, M. Cossi, J. M. Millam, M. Klene, C. Adamo, R. Cammi, J. W. Ochterski, R. L. Martin, K. Morokuma, O. Farkas, J. B. Foresman, D. J. Fox, *Gaussian 16 Rev. A.03*, Wallingford, CT, **2016**, available at

[5] Y. Zhao, D. G. Truhlar, *Theor. Chem. Acc.* **2008**, *120*, 215-241.

[6] D. Andrae, U. Häußermann, M. Dolg, H. Stoll, H. Preuß, *Theoretica chimica acta* **1990**, *77*, 123-141.

[7] W. R. Wadt, P. J. Hay, *J. Chem. Phys.* **1985**, *82*, 284-298.

Extraction of Different Metals

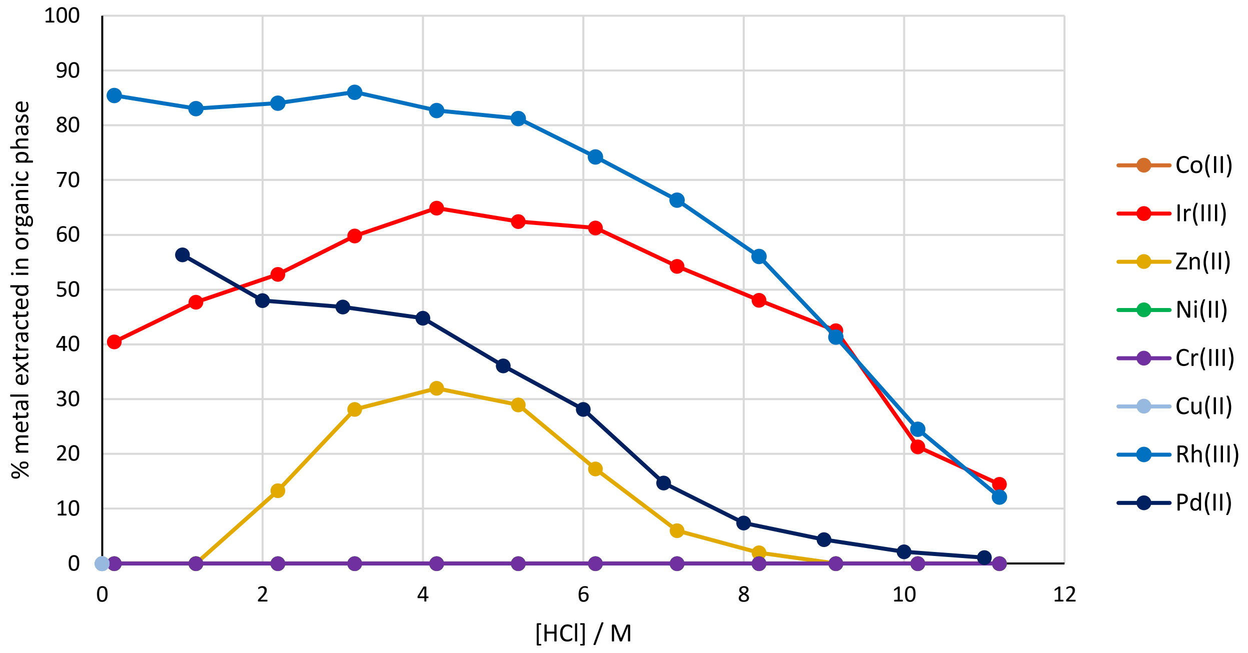

Figure S1: Extraction of metals from fresh aqueous solutions of varying HCl into a toluene solution of L^1/L^A. Conditions: Metal (0.01 M) in HCl (0 - 11 M, 2 mL), L^1 (0.1 M) and L^A (0.1 M) in toluene (2 mL), stirred for 1 h at RT.

Stripping Data

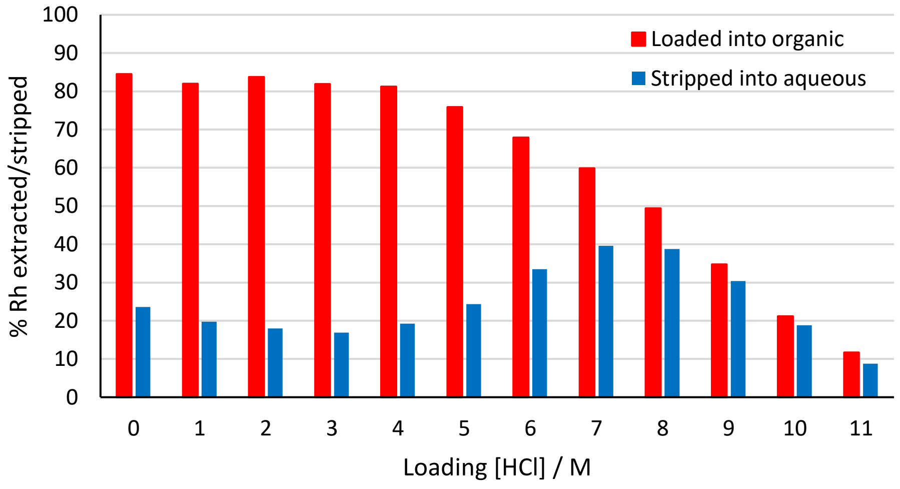

Figure S2: Loading of Rh into L^1 /L^A toluene solutions from fresh aqueous solution of varying [HCl], and the associated stripping performance using fresh water. Conditions (red bars): Rh (0.01 M) in fresh solutions of HCl (0 - 11 M, 2 mL), L^1 (0.1 M) and L^A (0.1 M) in toluene (2 mL), stirred for 1 h at RT. Stripping conditions (blue bars): Rh-loaded organic solution (1.5 mL), fresh water (1.5 mL), stirred for 1 h at RT.

Extractant Solubility Plots

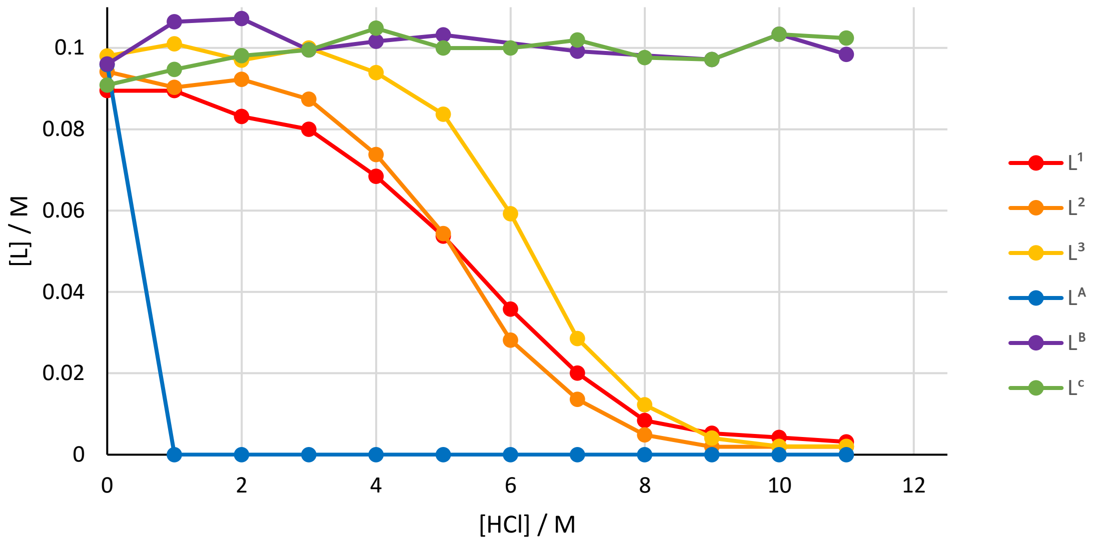

Figure S3: Concentrations of each of the amides and amines ($L^1/L^2/L^3/L^A/L^B/L^C$) in C_6D_6 (organic phase solubility) after contacting solutions containing a single extractant with aqueous solutions of varying [HCl]. Conditions: HCl (0 – 11 M, 2 mL), $L^1/L^2/L^3/L^A/L^B/L^C$ (0.1 M) in C_6D_6 (2 mL), stirred for 1 h at RT.

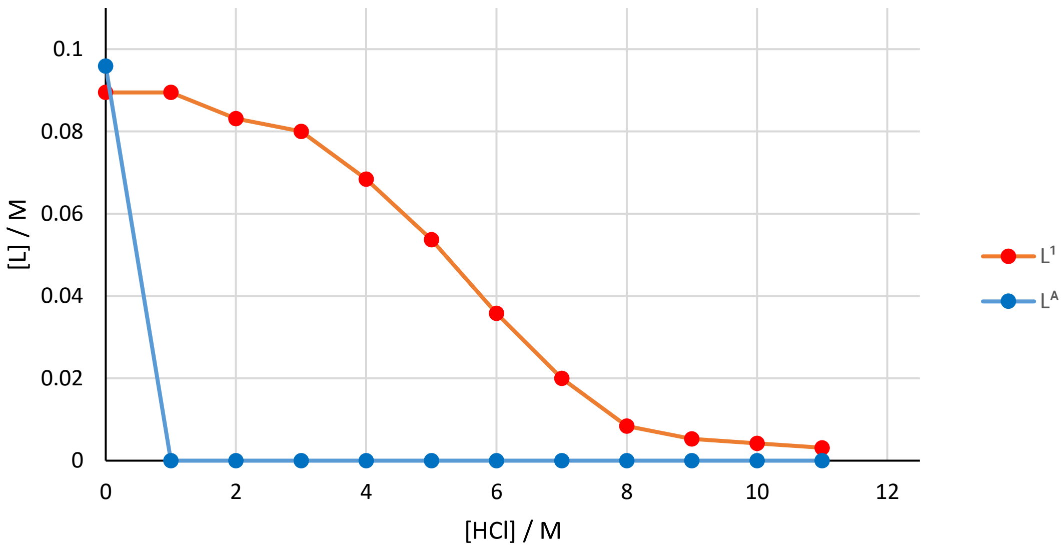

Figure S4: Concentrations of L^1 and L^A in C_6D_6 (organic phase solubility) after contacting a solution containing both extractants with aqueous solutions of varying [HCl]. Conditions: HCl (0 - 11 M, 2 mL), L^1 (0.1 M) and L^A (0.1 M) in C_6D_6 (2 mL), stirred for 1 h at RT.

KF Titration Data

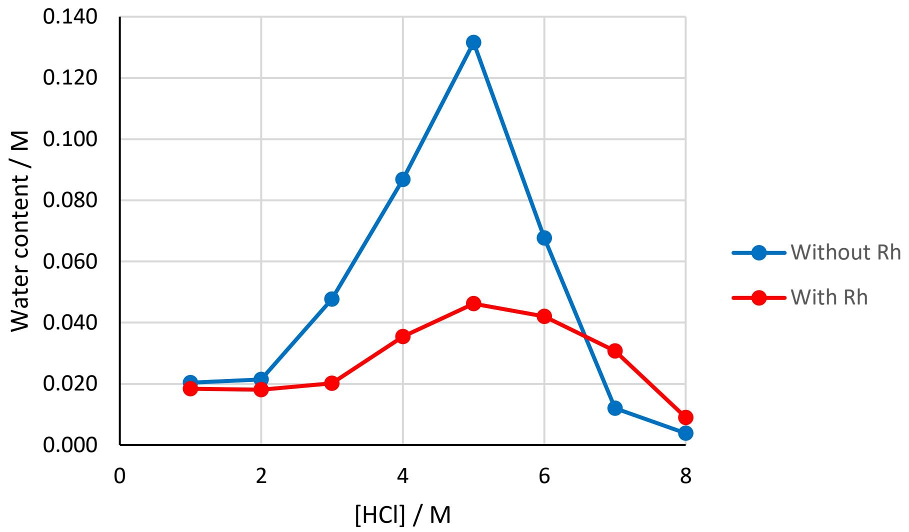

Figure S5: Water content by Karl-Fischer analysis in organic phases following extraction from varying [HCl] solutions with and without Rh. Conditions: Rh (0.01 M) in HCl (1 -8 M, 2 mL) aged for 1 day, or HCl (1 - 8 M, 2 mL) without Rh, L^1 (0.1 M) and L^A (0.1 M) in toluene (2 mL), stirred for 1 h at RT.

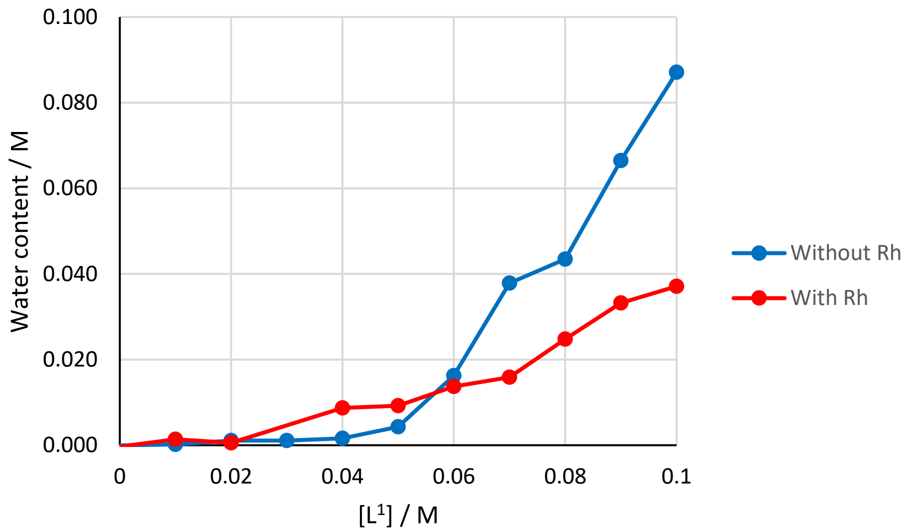

Figure S6: Water content by Karl-Fischer analysis in organic phases following extraction from HCl solutions with varying [L^1]. Conditions: Rh (0.01 M) in HCl (4 M, 2 mL) aged for 1 day, or HCl (4 M, 2 mL) without Rh, L^1 (0 - 0.1 M) and L^A (0.1 M) in toluene (2 mL), stirred for 1 h at RT.

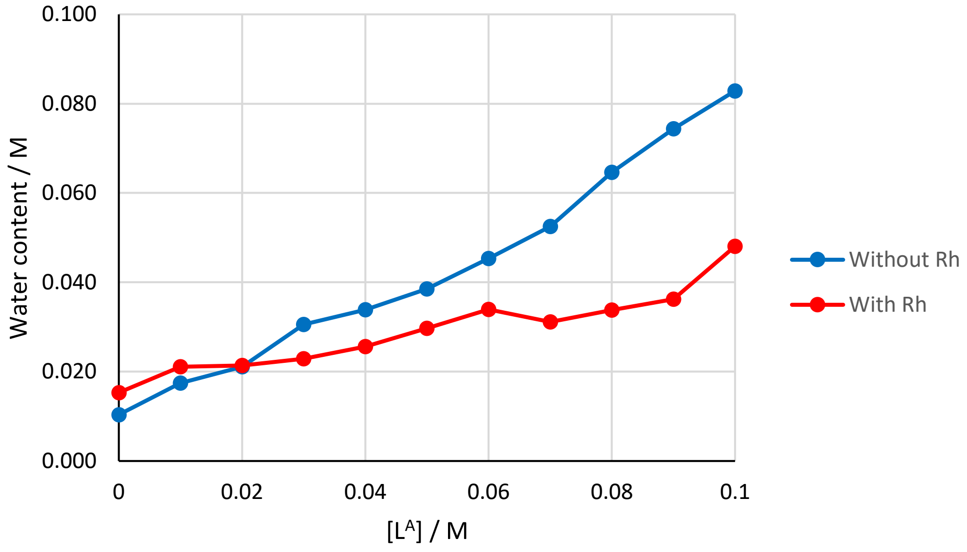

Figure S7: Water content by Karl-Fischer analysis in organic phases following extraction from HCl solutions with varying [L^A]. Conditions: Rh (0.01 M) in HCl (4 M, 2 mL) aged for 1 day prior to extraction, or HCl (4 M, 2mL) without Rh, L^1 (0.1 M) and L^A (0 - 0.1 M) in toluene (2 mL), stirred for 1 h at RT.

ESI-MS Spectra

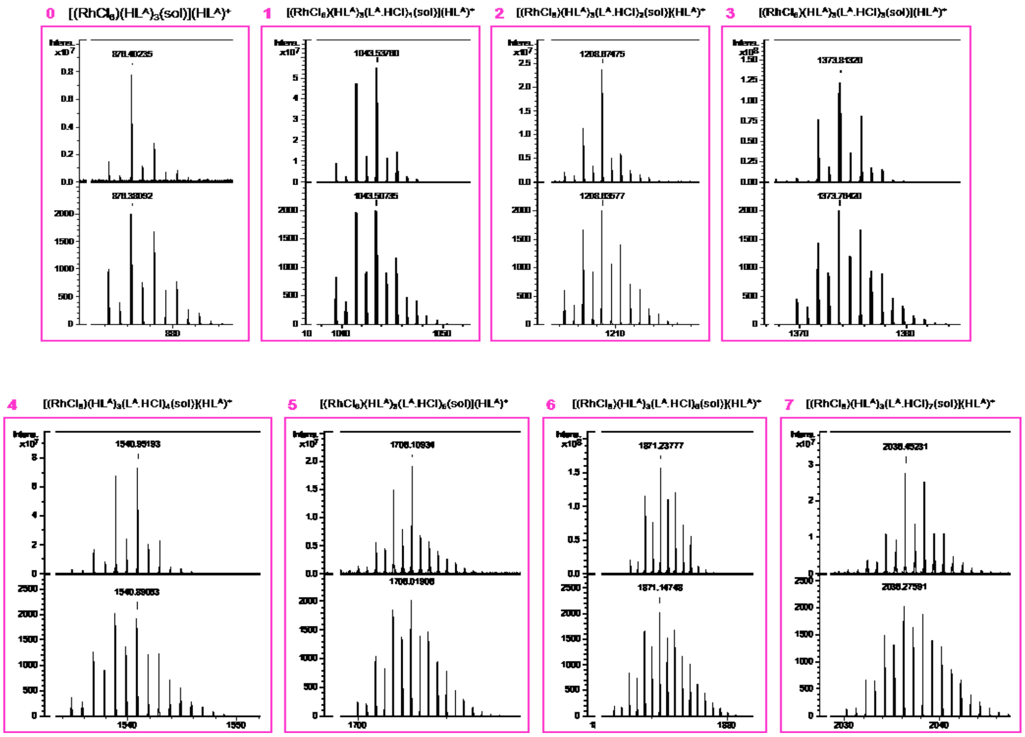

Figure S8: Observed (upper) and predicted (lower) isotopic distribution patterns for each assigned peak of the general formula $[(RhCl_6)(HL^A)_3(L^A.HCl)_{0-7}(sol)](HL^A)^+$. sol = CH_3CN.

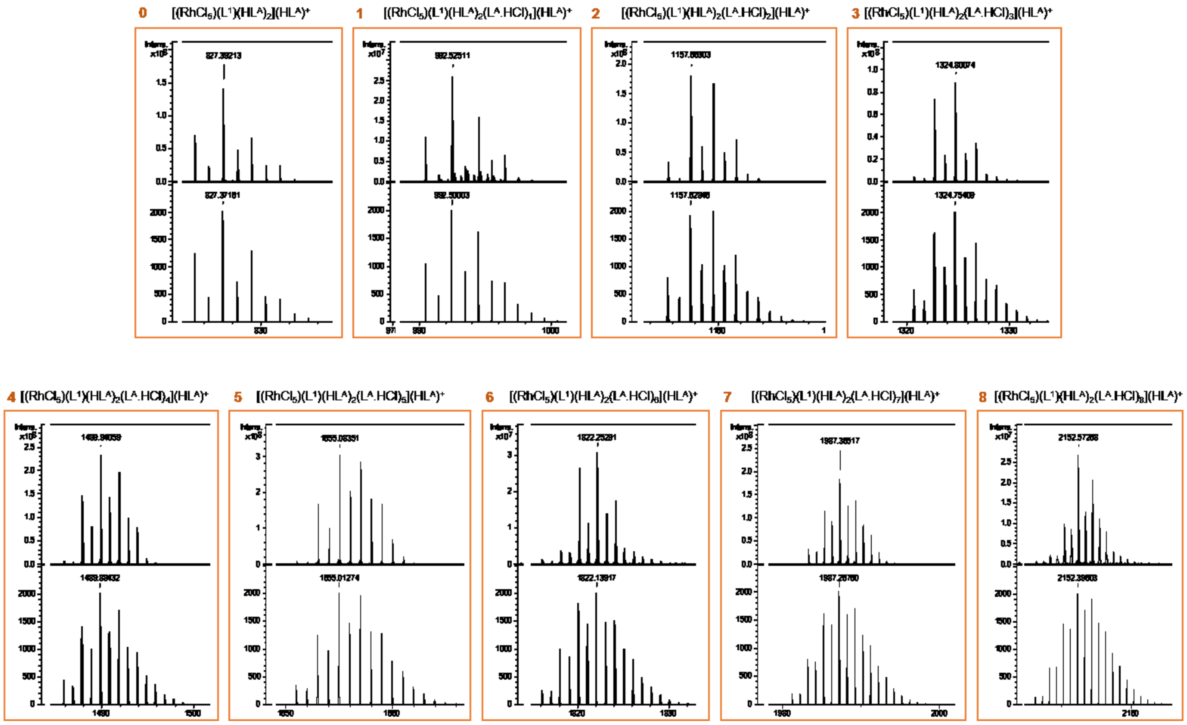

Figure S9: Observed (upper) and predicted (lower) isotopic distribution patterns for each assigned peak of the general formula $[(RhCl_5)(L^1)(HL^A)_2(L^A.HCl)_{0-8}](HL^A)^+$.

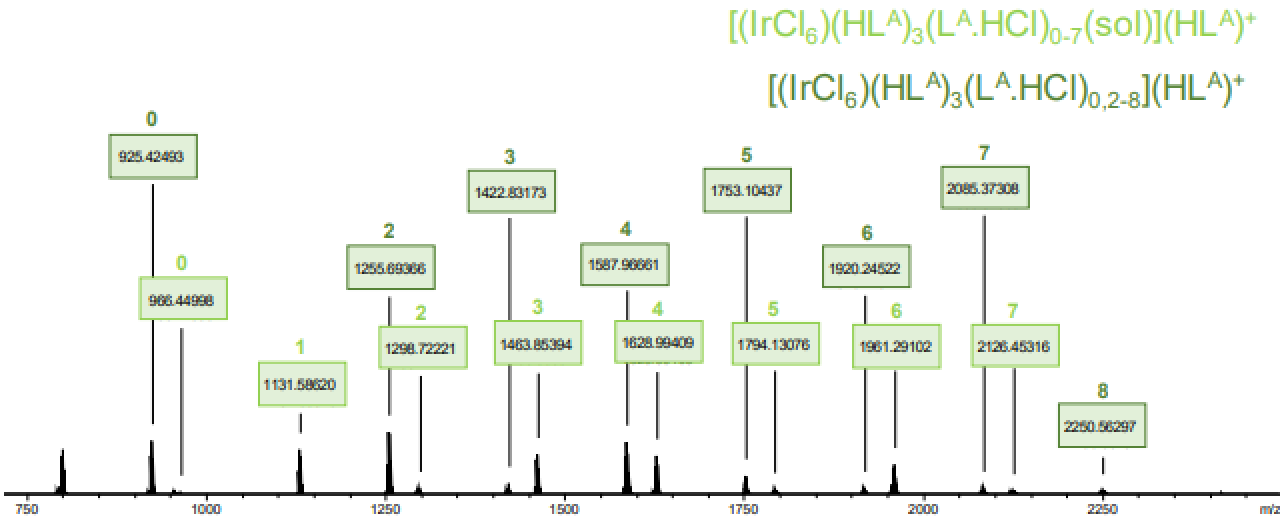

Figure S10: Positive-ion ESI-MS of an iridium-loaded L^1/L^A toluene solution diluted in CH_3CN. sol = CH_3CN.

Organic Phase Speciation

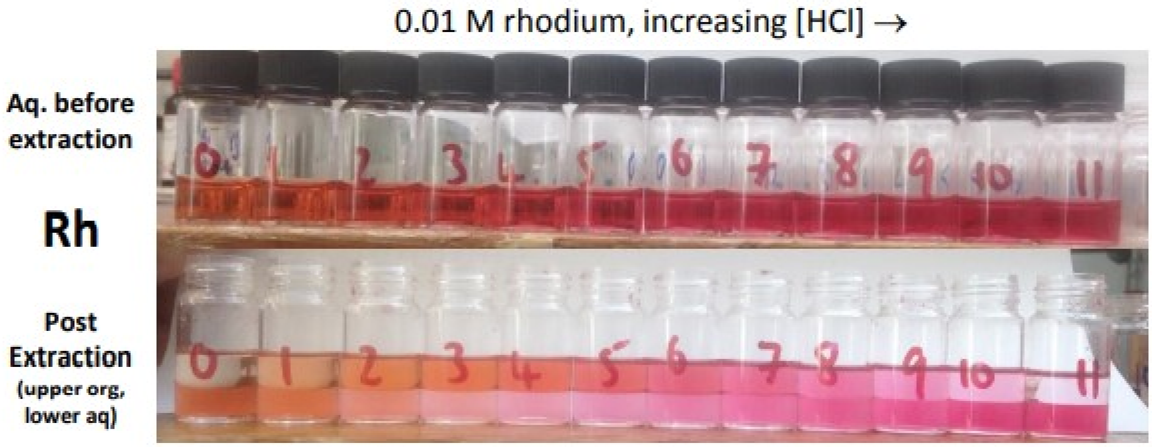

Figure S11: Photographs of rhodium-containing aqueous pre-extraction (upper) after a 1-day equilibration time, and post-extraction (lower), showing the variation in colour of solutions due to rhodium speciation.

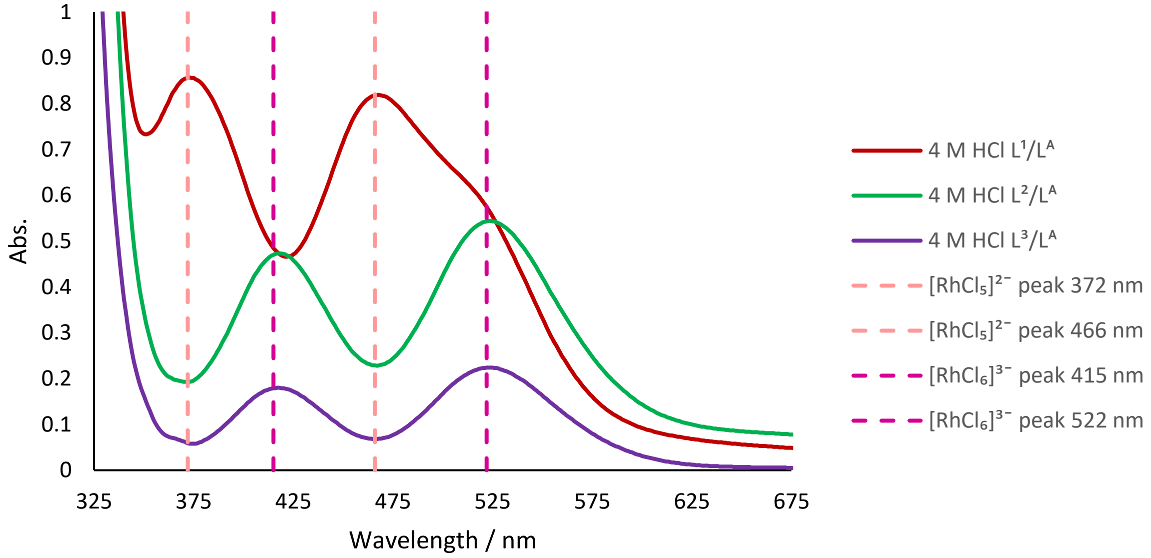

Figure S12: UV-vis spectra of organic phase containing L^A and L^1, L^2 or L^3 extractants after a 1 h contact with solutions of Rh in 4 M HCl. Conditions: Rh (0.01 M) in HCl (4 M, 2 mL) aged for 1 day, contacted with L^A (0.1 M) and L^1, L^2 or L^3 (0.1 M) in toluene (2 mL), stirred for 1 h at RT.

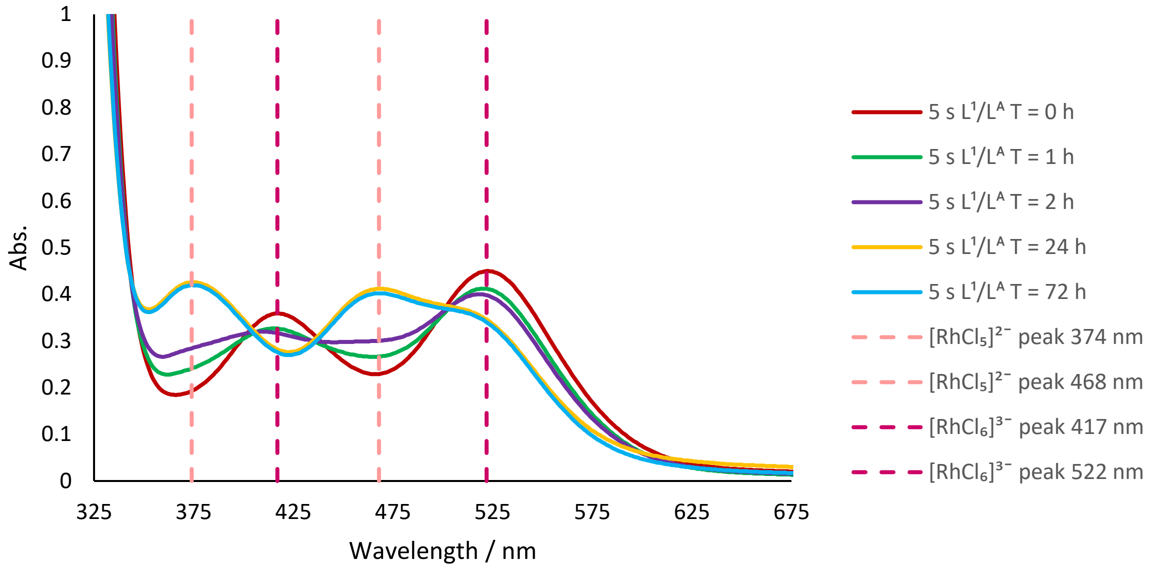

Figure S13: UV-vis spectra of organic phase containing both extractants after 5 s contact analysed between 0 and 72 h after extraction. Conditions: Rh (0.01 M) in HCl (4 M, 2 mL) aged for 1 day, L^1 (0.1 M) and L^A (0.1 M) in toluene (2 mL), stirred for 5 s at RT.

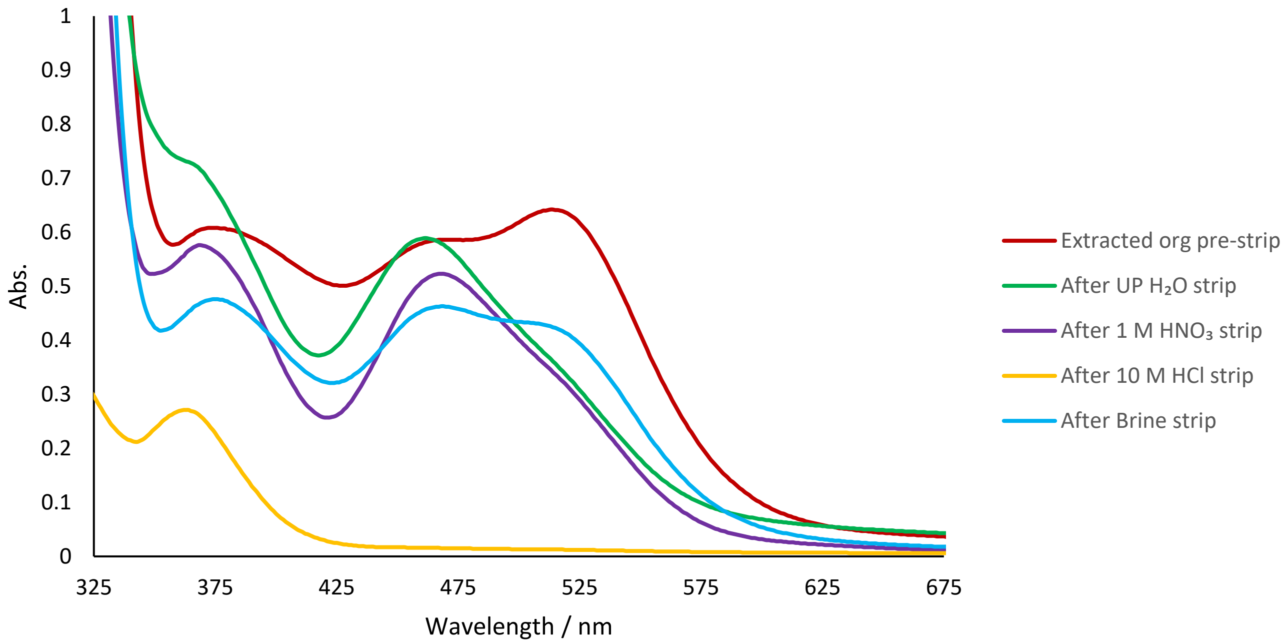

Figure S14: UV-vis spectra of post-extraction organic phase compared with UV-vis spectra of post-strip organic phases. Loading conditions: Rh (0.01 M) in HCl (6 M, 2 mL) aged for 1 day, L^1 (0.1 M) and L^A (0.1 M) in toluene (2 mL), stirred for 1 h at RT. Stripping conditions: Rh-loaded organic solution (1.5 mL), UP H_2O / 1 M HNO_3 / 10 M HCl / Brine (1.5 mL), stirred for 1 h at RT.

Crystallography of $[RhCl_6]^{3-}$ complex

Both amine molecules were modelled as completely disordered, one across a crystallographic mirror plane. Appropriate geometric (SAME) and displacement ellipsoid (RIGU) similarity restraints were used as outlined in the embedded res file. The water molecule was modelled as half occupied, located on a twofold rotation axis, such that there is one water per total formula ($RhCl_6^{3-}$, six protonated amines, three chlorides). N-bound H atoms were located from a difference Fourier map and refined using geometric restraints appropriate to a protonated primary amine.

Crystal Data. $C_{48}H_{122}Cl_9N_6ORh$, M_r = 1221.47, orthorhombic, Ibam (No. 72), a = 30.3256(4) Å, b = 9.82510(10) Å, c = 22.5098(3) Å, $\alpha = \beta = \gamma = 90°$, V = 6706.84(14) Å^3, T = 120.0 K, Z = 4, Z' = 0.25, $\mu(MoK_\alpha)$ = 0.648, 103086 reflections measured, 4258 unique (R_{int} = 0.0618) which were used in all calculations. The final wR_2 was 0.1545 (all data) and R_1 was 0.0585 (I > 2(I)).

Extraction of Rhodium from Varying Feed Concentrations

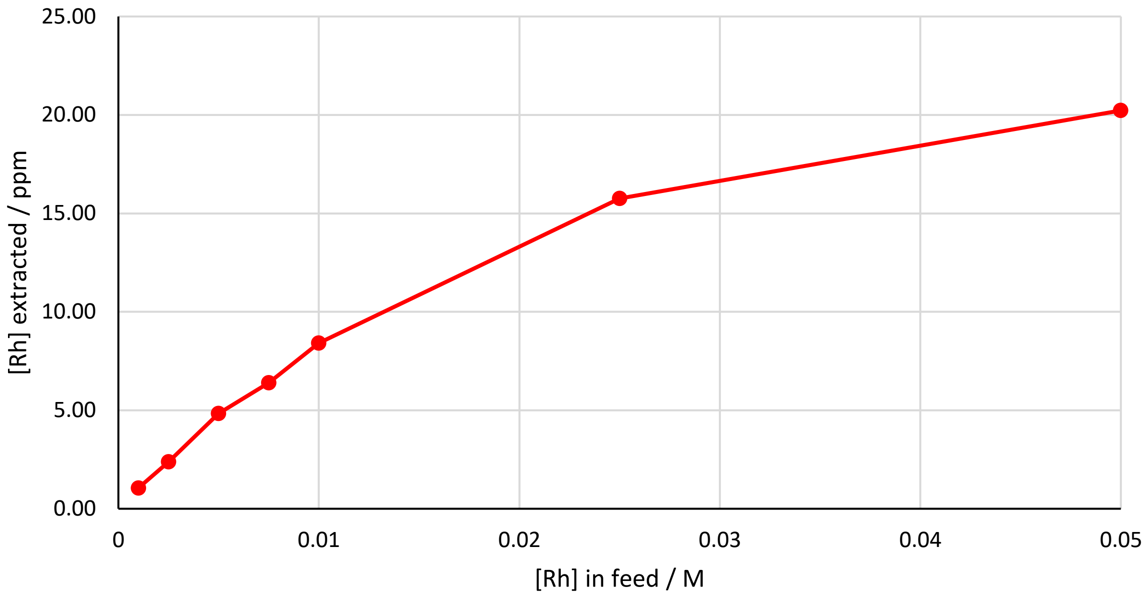

Figure S15: Extraction of Rh from aqueous solutions of varying Rh concentration. Conditions: Rh (0.001 - 0.05 M) in HCl (4 M, 2 mL) aged for 1 day, L^1 (0.1 M) and L^a (0.1 M) in toluene or C_6D_6 (2 mL), stirred for 1 h at RT.

Acid-Base Titration Data

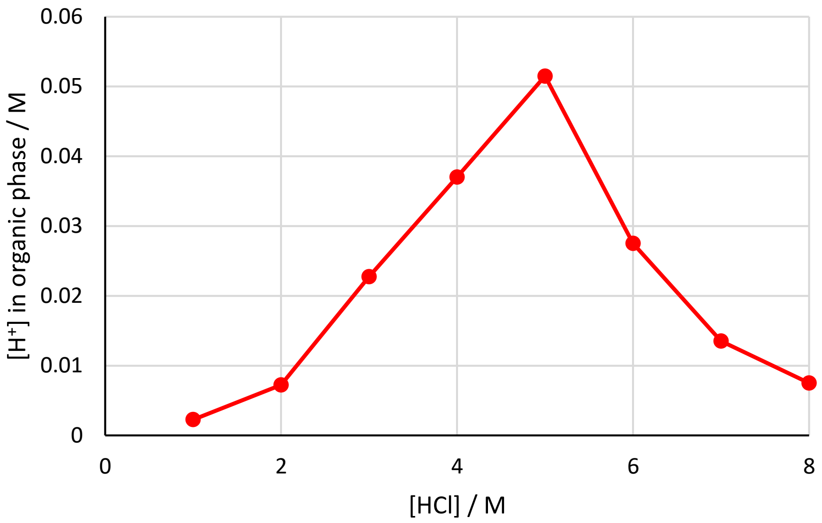

Figure S16: H^+ concentration by acid-base titration following extraction from varying [HCl] solutions without Rh. Conditions: HCl (1 - 8 M, 2 mL) without Rh, L^1 (0.1 M) and L^A (0.1 M) in toluene (2 mL), stirred for 1 h at RT.

Computational Data

Table S1: Gibbs free energies (kJmol^{-1}) of geometry-optimised structures, obtained from different starting configurations, and presented as relative values with respect to the lowest energy structure (dark green) in each case. Model complexity reduces from left to right, where the repeat energy values denote the same energy minimum has been obtained.

Complex	M06/6-31+G*		
	$[HL^D]_2[RhCl_5(L^4)](H_2O)$	$[HL^D]_2[RhCl_5(L^4)]$	$[RhCl_5(L^4)]$
N-bonded-amide-1	12.2	23.1	0.0
N-bonded-amide-2	19.8	16.2	0.0
N-bonded-amide-3	31.9	0.0	0.0
N-bonded-amide-4		7.0	0.0
N-bonded-enol-1	29.9	23.0	31.5
N-bonded-enol-2	38.8	24.7	31.5
N-bonded-enol-3	0.0	5.1	31.5
N-bonded-enol-4	31.1	50.8	79.8
N-bonded-enol-5	59.8		
O-bonded-amide-1	53.9	41.2	48.3
O-bonded-amide-2	49.3	41.2	48.3
O-bonded-amide-3	63.8	41.2	48.3
O-bonded-amide-4	44.0	25.9	48.3
O-bonded-enol-1	126.4	119.9	90.1
O-bonded-enol-2	137.9	116.6	90.2
O-bonded-enol-3	163.2	118.9	77.7
O-bonded-enol-4		129.8	90.2
Water-bound-1	64.8		
Water-bound-2	52.1		
Water-bound-3	37.2		
Water-bound-4			

$[HL^D]_2[RhCl_5(L^4)](H_2O)$: N-bonded-amide-1

Rh	-0.30686	-0.42991	-0.12833
Cl	-0.75789	1.80335	0.66817
Cl	-0.10972	-2.72588	-0.86242
Cl	0.74176	0.26440	-2.27590
Cl	1.83777	-0.29315	0.95644
Cl	-1.38736	-1.23217	1.80041
H	2.86062	-1.52936	-2.35401
H	1.90183	-2.51286	-1.42584
H	2.68496	-1.28391	-0.71213
C	2.18796	-4.74932	1.67350
H	2.94767	-4.92605	2.44854
C	2.80851	-4.59462	0.29410
H	1.99449	-4.39386	-0.41891
H	3.27847	-5.53760	-0.03592
H	4.88980	-2.24321	-1.22051
N	2.82302	-2.01338	-1.45253
H	1.63020	-3.84115	1.94377
C	3.86494	-3.48304	0.24731
C	5.22741	-3.99436	0.69868
H	5.62481	-4.73072	-0.01717
H	5.15495	-4.49272	1.67336
C	3.99588	-2.87631	-1.14376
H	4.06153	-3.65672	-1.91535
H	3.54585	-2.67521	0.93059
H	1.48295	-5.58874	1.70180
H	2.08736	1.83562	-1.20257
H	1.25398	2.40112	0.07676
H	1.16924	3.20376	-1.34743
H	2.09558	2.82783	2.30084
H	2.88220	4.24166	3.03892
C	2.56152	4.87709	0.26753
H	2.07289	5.42592	-0.55769
C	3.79555	5.66421	0.69261
H	4.35576	5.14241	1.48146
H	3.51283	6.64989	1.08360
C	3.00682	3.53219	-0.28927
H	3.56002	2.94708	0.45847
N	1.84974	2.71486	-0.71758
C	1.95242	3.88300	2.57581
H	1.17545	3.89002	3.34927
C	1.52921	4.74642	1.39495
H	0.58040	4.36001	0.98719
H	3.66181	3.67183	-1.15896
H	1.29702	5.76385	1.74562
N	-2.16628	-0.56512	-1.16600
C	-3.00456	0.60311	-1.08323
C	-3.90598	0.65718	0.10971
C	-4.94701	-0.47028	0.16567
C	-5.76225	-0.31576	1.44953
C	-6.76722	-1.43213	1.68640
O	-2.92947	1.43854	-1.95484
C	-5.81862	-0.47686	-1.08401
H	-7.57636	-1.42640	0.94391
H	-6.27949	-2.41684	1.64269
H	-7.23377	-1.33960	2.67513
H	-3.27493	0.61371	1.01109
H	-4.40704	1.63527	0.08190
H	-4.41836	-1.43924	0.24337
H	-5.22408	-0.57028	-2.00481
H	-6.38762	0.46294	-1.16004
H	-6.53620	-1.30683	-1.07797
H	-6.28122	0.65847	1.42448
H	-5.06004	-0.26976	2.29696
H	4.48288	5.82460	-0.14962
H	5.96180	-3.18297	0.79443
H	-2.57477	-1.40782	-0.75289
O	-0.45510	3.55285	-2.07255
H	-1.00050	3.17690	-1.35344
H	-0.61170	2.92879	-2.79886
H	-1.86634	-0.73380	-2.13017

$[HL^D]_2[RhCl_5(L^4)](H_2O)$: N-bonded-enol-3

Rh	-0.11466	-0.24032	-0.04314
Cl	-1.72803	-0.32136	-1.82592
Cl	1.56551	0.12174	1.67328
Cl	-1.82095	0.34031	1.62582
Cl	-0.46996	-2.63201	0.45506
Cl	1.59077	-0.81473	-1.59658
H	2.26765	-2.95702	1.76344
H	2.71733	-1.63584	0.90719
H	1.98539	-2.85561	0.12219
C	5.52244	-0.56266	-1.76709
H	6.42365	-1.01923	-2.19776
C	5.07514	-1.25723	-0.49041
H	4.23936	-0.67625	-0.06902
H	5.88444	-1.23459	0.26128
H	3.84522	-4.37031	0.45771
N	2.69450	-2.67401	0.85123
H	4.72622	-0.59734	-2.52388
C	4.65050	-2.71274	-0.70212
C	5.82745	-3.59897	-1.09209
H	6.59694	-3.59519	-0.30408
H	6.30146	-3.25442	-2.01836
C	4.01030	-3.28964	0.55346
H	4.65557	-3.12620	1.42866
H	3.89764	-2.73779	-1.51067
H	5.75100	0.49348	-1.57856
H	-2.87461	-2.74641	0.28030
H	-3.09826	-1.43613	-0.68733
H	-3.12122	-1.25264	0.92891
H	-4.96312	-1.17835	-2.46804
H	-6.63523	-0.58357	-2.37908
C	-5.68040	-0.80721	0.32297
H	-5.37979	-0.33862	1.27734
C	-7.17227	-1.11198	0.39596
H	-7.52032	-1.64929	-0.49779
H	-7.75465	-0.18490	0.47250
C	-4.92095	-2.12237	0.21212
H	-5.19522	-2.67208	-0.69825
N	-3.45519	-1.90188	0.18313
C	-5.58480	-0.30822	-2.21132
H	-5.32889	0.47375	-2.93610
C	-5.33628	0.18673	-0.79335
H	-4.28450	0.50343	-0.70266
H	-5.14325	-2.76855	1.07083
H	-5.92907	1.09693	-0.61435
N	0.27848	1.68030	-0.55639
C	-0.38534	2.78062	-0.50081
C	0.23262	4.09283	-0.87237
C	1.29388	4.52800	0.15261
C	1.84936	5.89133	-0.25814
C	3.02693	6.36164	0.58168
O	-1.61930	2.86464	-0.05692
C	0.72264	4.52847	1.56484
H	2.73644	6.56672	1.62008
H	3.82369	5.60403	0.60369
H	3.45882	7.28539	0.17650
H	-1.90354	2.00288	0.34475
H	0.68511	4.01670	-1.87179
H	-0.56736	4.84544	-0.92214
H	2.12256	3.79591	0.11845
H	0.37153	3.53115	1.86543
H	-0.12907	5.22288	1.63879
H	1.47593	4.83398	2.30123
H	1.03408	6.63434	-0.20908
H	2.15408	5.84497	-1.31670
H	-7.41613	-1.73200	1.26950
H	5.51562	-4.64004	-1.25257
H	1.22798	1.76322	-0.91898
O	1.14656	-2.73481	3.20736
H	0.33639	-2.85370	2.67446
H	1.20520	-1.76299	3.27202

$[HL^D]_2[RhCl_5(L^4)](H_2O)$: O-bonded-amide-4

Rh	-0.31554	-0.19963	-0.35464
Cl	0.12388	-2.29309	-1.43389
Cl	-1.04118	1.83643	0.77079
Cl	1.09802	-0.79848	1.69037
Cl	1.62665	0.81219	-1.36069
Cl	-1.58418	0.42502	-2.20942
H	1.98478	1.46715	2.00819
H	0.89884	2.48356	1.31575
H	2.11430	1.88378	0.41968
C	1.63424	5.85537	-1.11388
H	2.01936	6.83641	-0.80388
C	1.55706	4.86536	0.03687
H	0.96542	4.00931	-0.32295
H	0.99609	5.30722	0.88175
H	3.75899	3.04462	2.02547
N	1.92202	2.27416	1.37081
H	2.28468	5.46866	-1.91247
C	2.92101	4.39628	0.54420
C	3.81549	5.54975	0.98100
H	3.30668	6.17019	1.73526
H	4.06853	6.19967	0.13580
C	2.78084	3.43492	1.71587
H	2.32811	3.93821	2.58228
H	3.42214	3.85236	-0.27946
H	0.64083	5.99289	-1.55456
H	3.21244	-1.07055	-1.13339
H	2.09920	-2.26701	-0.97505
H	2.69065	-1.54435	0.36016
H	2.35028	-4.71217	-1.81041
H	3.04653	-6.17519	-1.07976
C	3.78604	-3.95240	0.59245
H	3.72146	-3.42031	1.55920
C	4.95599	-4.92672	0.66622
H	5.11948	-5.43580	-0.29424
H	4.76989	-5.70001	1.42245
C	4.08890	-2.90727	-0.47271
H	4.20657	-3.36315	-1.46480
N	3.00236	-1.90294	-0.56611
C	2.28947	-5.38999	-0.94665
H	1.30449	-5.86804	-1.00495
C	2.44133	-4.65871	0.37977
H	1.61700	-3.93615	0.49117
H	5.02069	-2.37640	-0.23830
H	2.31435	-5.37298	1.20790
O	-2.08239	-1.11085	0.28912
C	-2.65058	-1.13075	1.39766
C	-4.09825	-1.53439	1.41704
C	-4.93153	-0.70999	0.42959
C	-6.36348	-1.24291	0.41763
C	-7.26045	-0.58570	-0.62018
N	-2.05508	-0.82366	2.55053
C	-4.86164	0.77160	0.77791
H	-7.44576	0.47208	-0.39271
H	-6.80537	-0.63463	-1.61964
H	-8.23713	-1.08345	-0.67350
H	-1.07225	-0.55003	2.55459
H	-2.58034	-0.79319	3.41222
H	-4.14347	-2.60012	1.14571
H	-4.51187	-1.43817	2.43490
H	-4.48973	-0.85338	-0.57080
H	-3.82868	1.14871	0.76364
H	-5.28452	0.95635	1.78015
H	-5.42469	1.38041	0.06006
H	-6.80257	-1.11543	1.42395
H	-6.33452	-2.32977	0.23405
H	5.89224	-4.41725	0.93307
H	4.75979	5.19408	1.41736
O	-0.72984	3.67638	-2.01899
H	-0.55657	2.90229	-2.57804
H	-1.20507	3.27396	-1.27265

$[HL^D]_2[RhCl_5(L^4)](H_2O)$: O-bonded-enol-1

Rh	-0.04324	-0.24857	-0.27752
Cl	1.58186	-1.70548	-1.34945
Cl	-1.82613	0.97093	0.74711
Cl	1.44483	0.24832	1.65093
Cl	0.81472	1.67199	-1.39459
Cl	-1.50490	-0.62823	-2.09619
H	0.75654	2.90206	2.15574
H	-0.65653	2.56361	1.34539
H	0.69914	2.79602	0.49578
C	-2.96856	4.11914	-1.26148
H	-2.87417	4.92976	-1.99847
C	-2.33709	4.48675	0.07178
H	-2.43590	3.61930	0.74183
H	-2.88843	5.31138	0.55618
H	0.89977	5.13804	1.18677
N	0.20514	3.16832	1.33504
H	-2.48705	3.22163	-1.67583
C	-0.86713	4.90463	-0.06874
C	-0.74306	6.38155	-0.42234
H	-1.09000	7.01242	0.41071
H	-1.35898	6.62738	-1.29623
C	-0.07127	4.62538	1.19989
H	-0.61910	4.95289	2.09469
H	-0.41465	4.30715	-0.88133
H	-4.03616	3.89608	-1.14840
H	3.31041	1.17417	-1.18838
H	3.19497	-0.45169	-0.98095
H	3.09716	0.52207	0.31787
H	4.94470	-2.19606	-1.66190
H	6.37481	-2.85546	-0.84046
C	5.44807	-0.64307	0.74204
H	4.99781	-0.24675	1.66988
C	6.96220	-0.65590	0.91802
H	7.47408	-0.97877	0.00031
H	7.25296	-1.34696	1.71931
C	5.09793	0.32946	-0.37609
H	5.54976	0.02924	-1.33093
N	3.62971	0.41295	-0.57646
C	5.28530	-2.73490	-0.76541
H	4.83967	-3.73504	-0.82283
C	4.86649	-2.04568	0.52580
H	3.76667	-2.00810	0.57897
H	5.46081	1.33768	-0.13859
H	5.17377	-2.66138	1.38476
O	-0.70884	-2.05356	0.68912
C	-1.54836	-2.22399	1.78994
C	-2.72543	-3.09363	1.48653
C	-3.77319	-2.34895	0.63872
C	-4.65555	-3.35119	-0.10248
C	-5.67134	-2.69750	-1.02642
N	-1.32113	-1.67869	2.90251
C	-4.57568	-1.39988	1.52002
H	-6.42763	-2.12915	-0.46848
H	-5.17365	-1.99919	-1.71626
H	-6.20196	-3.44436	-1.63113
H	-0.48034	-1.09405	2.85946
H	-2.38584	-3.99472	0.95085
H	-3.16616	-3.41675	2.43992
H	-3.24436	-1.74559	-0.12150
H	-3.91409	-0.79449	2.15350
H	-5.25080	-1.96885	2.18040
H	-5.18393	-0.70797	0.92297
H	-5.17114	-3.99221	0.63574
H	-4.00651	-4.02053	-0.69100
H	7.34894	0.33881	1.17920
H	0.29241	6.66556	-0.65486
H	-0.90616	-2.71549	-0.03856
O	-1.06608	-3.66157	-1.42422
H	-0.13270	-3.72871	-1.68491
H	-1.39583	-2.91545	-1.96890

$[HL^D]_2[RhCl_5(L^4)](H_2O)$: Water-bound-3

Rh	-0.43699	-0.27906	0.34413
Cl	-1.57592	-2.14460	1.37022
Cl	0.99687	1.36812	-0.67251
Cl	-1.79100	-0.39450	-1.73499
Cl	-1.98778	1.33426	1.23509
Cl	0.88103	-0.15506	2.29217
H	-1.95341	2.25174	-2.36328
H	-0.57929	2.46056	-1.45620
H	-2.00137	2.29662	-0.70089
C	0.82508	4.81579	1.18114
H	0.40213	5.58909	1.83871
C	0.25897	4.89575	-0.22771
H	0.69356	4.07038	-0.81146
H	0.57699	5.82637	-0.72921
H	-2.88616	4.38676	-1.64281
N	-1.58626	2.74920	-1.54650
H	0.60371	3.83345	1.62348
C	-1.27438	4.83276	-0.24711
C	-1.88701	6.21484	-0.05773
H	-1.66741	6.86079	-0.92207
H	-1.47381	6.70751	0.83137
C	-1.80590	4.22013	-1.53676
H	-1.30615	4.64806	-2.41743
H	-1.60206	4.18382	0.58528
H	1.91487	4.94090	1.18049
H	-4.08439	0.01967	0.86666
H	-3.45258	-1.49426	0.78531
H	-3.55606	-0.60555	-0.57170
H	-4.60472	-3.67549	1.47236
H	-5.68074	-4.79834	0.61333
C	-5.38738	-2.47380	-1.05417
H	-5.01640	-1.99716	-1.97929
C	-6.80130	-2.97983	-1.31533
H	-7.25349	-3.40482	-0.40784
H	-6.79675	-3.76551	-2.08156
C	-5.44823	-1.38980	0.01355
H	-5.85577	-1.77546	0.95781
N	-4.10308	-0.83400	0.29334
C	-4.68464	-4.33463	0.59550
H	-3.94755	-5.13224	0.74638
C	-4.40903	-3.60402	-0.71147
H	-3.37805	-3.21600	-0.70100
H	-6.09229	-0.56320	-0.31332
H	-4.43691	-4.32269	-1.54477
O	3.48091	-1.21719	-0.48837
C	4.22736	-0.35308	-0.01314
C	5.63578	-0.20411	-0.53653
C	6.40631	-1.52541	-0.51777
C	7.75293	-1.34392	-1.21657
C	8.56237	-2.62459	-1.35284
N	3.85303	0.47034	0.97819
C	6.56182	-2.02965	0.91184
H	8.89584	-3.00622	-0.37879
H	7.96854	-3.41588	-1.83322
H	9.45992	-2.46592	-1.96423
H	2.89210	0.44014	1.33444
H	4.46953	1.19411	1.31776
H	5.55474	0.16208	-1.57172
H	6.19363	0.55712	0.03454
H	5.81064	-2.25846	-1.08759
H	5.58967	-2.13023	1.41334
H	7.17586	-1.33132	1.50416
H	7.04655	-3.01335	0.94865
H	8.34229	-0.58797	-0.66687
H	7.57463	-0.91929	-2.21814
H	-7.46172	-2.17501	-1.66687
H	-2.97791	6.17004	0.06398
O	0.85793	-1.73639	-0.42178
H	1.81325	-1.43290	-0.41098
H	0.80366	-2.46265	0.22404

[HL^D]$_2$[RhCl$_5$(L^4)]: N-bonded-amide-3

Rh	-0.05553	0.08882	0.47345
Cl	-1.52282	-0.26012	-1.40348
Cl	1.36842	0.57744	2.36770
Cl	-1.31805	-1.36774	1.92658
Cl	1.33908	-1.73708	-0.29048
Cl	1.15542	1.70393	-0.84407
H	2.97632	0.68258	1.01190
H	3.96402	1.05778	-0.29300
H	2.96375	-0.28304	-0.29902
C	6.87061	-1.33701	-2.22817
H	7.75005	-1.82631	-1.79072
C	6.08516	-0.54631	-1.19249
H	5.33032	0.05528	-1.72491
H	6.75509	0.17042	-0.68349
H	4.12818	-1.20788	1.61433
N	3.61545	0.28750	0.28086
H	6.24459	-2.11747	-2.68389
C	5.40003	-1.41622	-0.13614
C	6.38877	-2.30859	0.60440
H	7.19927	-1.70928	1.04806
H	6.84741	-3.04619	-0.06397
C	4.65921	-0.57476	0.89153
H	5.34961	0.07434	1.44917
H	4.65480	-2.05765	-0.64170
H	7.22669	-0.68561	-3.03520
H	-0.49055	-3.50419	-0.79794
H	-1.62648	-2.33351	-1.04959
H	-1.37122	-2.87631	0.46097
H	-3.71008	-2.48495	-2.54851
H	-5.34542	-3.13203	-2.29468
C	-3.77839	-3.88049	-0.00069
H	-3.55143	-3.79650	1.07682
C	-4.77940	-5.01540	-0.18716
H	-4.99657	-5.19398	-1.25019
H	-5.72925	-4.77796	0.30863
C	-2.48166	-4.26417	-0.70171
H	-2.63255	-4.41044	-1.77991
N	-1.43855	-3.22200	-0.52639
C	-4.61921	-2.38880	-1.93631
H	-5.02846	-1.39494	-2.15338
C	-4.33678	-2.52334	-0.44667
H	-3.66084	-1.71395	-0.12796
H	-2.08586	-5.20115	-0.28946
H	-5.26660	-2.35393	0.11744
N	-1.23678	1.67678	1.24435
C	-2.38512	2.10469	0.46949
C	-2.08657	3.03767	-0.66358
C	-1.45689	4.37100	-0.23722
C	-1.16836	5.19694	-1.49078
C	-0.43427	6.50104	-1.22065
O	-3.47786	1.71014	0.79100
C	-2.34828	5.10884	0.75433
H	-1.04814	7.21286	-0.65275
H	0.48660	6.32152	-0.64650
H	-0.14786	6.99525	-2.15767
H	-1.41070	2.52267	-1.36236
H	-3.04200	3.21870	-1.17633
H	-0.47767	4.16603	0.23513
H	-2.57140	4.50262	1.64461
H	-3.31262	5.36340	0.28745
H	-1.88682	6.04031	1.10579
H	-2.12226	5.40232	-2.00753
H	-0.57147	4.57724	-2.17848
H	-4.40824	-5.95854	0.23747
H	5.89752	-2.86235	1.41539
H	-0.53341	2.40851	1.37748
H	-1.55320	1.31252	2.14821

$[HL^D]_2[RhCl_5(L^4)]$: N-bonded-enol-3

Rh	0.50483	-0.16194	0.05700
Cl	0.07445	-2.23887	1.20429
Cl	1.13950	1.89182	-1.04366
Cl	0.85539	-1.34318	-2.04958
Cl	-1.87028	0.09005	-0.54912
Cl	0.17715	1.01219	2.11480
H	-0.35096	2.84869	0.07931
H	-1.36662	3.36069	1.31695
H	-1.72453	2.02614	0.37041
C	-5.52421	4.23752	1.10877
H	-6.02574	4.99146	0.48888
C	-4.01675	4.24188	0.90307
H	-3.56591	3.59585	1.67393
H	-3.61977	5.25906	1.07490
H	-1.75852	3.42444	-1.62098
N	-1.35014	3.00086	0.36087
H	-5.95188	3.25683	0.85540
C	-3.57786	3.76848	-0.48414
C	-4.20471	4.59455	-1.60082
H	-3.99360	5.66623	-1.45941
H	-5.29327	4.47118	-1.63510
C	-2.06644	3.82351	-0.64550
H	-1.69409	4.85523	-0.56700
H	-3.88736	2.71431	-0.60678
H	-5.78081	4.45021	2.15354
H	-1.29985	-2.19680	-1.79610
H	-1.24220	-2.79560	-0.28238
H	-1.23713	-3.83869	-1.58189
H	-3.17537	-1.86135	1.37729
H	-4.70602	-2.63017	1.87224
C	-3.66253	-4.17765	-0.31068
H	-3.25304	-5.12931	-0.69917
C	-5.18117	-4.24264	-0.43523
H	-5.64784	-3.28952	-0.15009
H	-5.59211	-5.02223	0.21917
C	-3.15685	-3.05592	-1.20478
H	-3.49946	-2.07145	-0.86281
N	-1.67141	-2.98134	-1.22833
C	-3.61423	-2.75382	1.84648
H	-3.25752	-2.75875	2.88335
C	-3.20236	-4.04224	1.14707
H	-2.10647	-4.14364	1.20783
H	-3.49569	-3.19181	-2.24006
H	-3.60362	-4.90801	1.69721
N	2.44567	-0.27538	0.63086
C	3.42728	-1.05519	0.34292
C	4.81369	-0.79369	0.84511
C	5.42841	0.45034	0.18136
C	6.85753	0.62927	0.69264
C	7.52182	1.92183	0.24394
O	3.32344	-2.09204	-0.45671
C	5.35616	0.35209	-1.33724
H	7.69045	1.94345	-0.84045
H	6.90235	2.79343	0.50055
H	8.49792	2.05466	0.72744
H	4.79224	-0.66446	1.93704
H	5.42969	-1.67702	0.62324
H	4.83881	1.33193	0.49583
H	4.31922	0.27461	-1.69386
H	5.90365	-0.53403	-1.69534
H	5.79072	1.23777	-1.81669
H	7.46077	-0.23538	0.36447
H	6.84657	0.59075	1.79449
H	-5.49351	-4.46893	-1.46392
H	-3.81121	4.30142	-2.58320
H	2.67214	0.49245	1.26251
H	2.43371	-2.10311	-0.89803

[HL^D]$_2$[$RhCl_5(L^4)$]: O-bonded-amide-4

Rh	0.52625	-0.25256	0.05349
Cl	-0.01896	-2.27576	1.26125
Cl	1.24947	1.71843	-1.12596
Cl	0.84328	-1.52552	-2.01626
Cl	-1.76313	0.10390	-0.57525
Cl	0.18531	0.97558	2.07014
H	-0.15257	2.79200	-0.06452
H	-1.02855	3.49980	1.18571
H	-1.53514	2.10036	0.43739
C	-5.13514	4.62125	1.21204
H	-5.65141	5.32536	0.54718
C	-3.65346	4.51085	0.88482
H	-3.16876	3.93329	1.68873
H	-3.19232	5.51520	0.89920
H	-1.66904	3.27546	-1.69967
N	-1.11720	3.03886	0.27814
H	-5.63192	3.64495	1.11714
C	-3.36266	3.85466	-0.46649
C	-4.03968	4.58502	-1.61991
H	-3.75825	5.64980	-1.62632
H	-5.13216	4.52971	-1.55033
C	-1.87025	3.79393	-0.75308
H	-1.43510	4.80171	-0.81827
H	-3.73795	2.81499	-0.43728
H	-5.28749	4.97095	2.24019
H	-1.26185	-2.31421	-1.77751
H	-1.34207	-2.81058	-0.22831
H	-1.39114	-3.93549	-1.45584
H	-3.24604	-1.63270	1.31377
H	-4.85822	-2.24056	1.77372
C	-3.87940	-3.96881	-0.30183
H	-3.55144	-4.97332	-0.63066
C	-5.39131	-3.89324	-0.48667
H	-5.77227	-2.88683	-0.26421
H	-5.90125	-4.59677	0.18416
C	-3.23172	-2.94623	-1.22338
H	-3.49201	-1.91816	-0.94322
N	-1.74723	-3.01587	-1.18055
C	-3.78191	-2.46184	1.79797
H	-3.46633	-2.45475	2.84808
C	-3.46272	-3.81036	1.16678
H	-2.38327	-4.00531	1.27645
H	-3.53964	-3.10209	-2.26551
H	-3.96045	-4.61266	1.73428
O	2.51594	-0.36595	0.68134
C	3.40139	-1.22013	0.48981
C	4.79948	-0.85024	0.89722
C	5.25811	0.42115	0.17097
C	6.66530	0.78786	0.63897
C	7.17111	2.11763	0.10081
C	5.17166	0.23608	-1.33913
H	7.31978	2.09168	-0.98659
H	6.45799	2.92565	0.31833
H	8.13251	2.39059	0.55446
H	4.79981	-0.68227	1.98383
H	5.49779	-1.67755	0.68782
H	4.56401	1.22831	0.46010
H	4.14532	0.01180	-1.66381
H	5.82840	-0.58637	-1.67020
H	5.47600	1.14322	-1.87548
H	7.36158	-0.02013	0.34989
H	6.67426	0.81642	1.74104
H	-5.68571	-4.13791	-1.51648
H	-3.75092	4.15551	-2.58838
H	2.28320	-2.60009	-0.49831
H	3.97101	-3.00811	-0.30489
N	3.18995	-2.41390	-0.06663

[$HL^D]_2[RhCl_5(L^4)]$: O-bonded-enol-2

Rh	0.37050	-0.68167	-0.31357
Cl	-0.47229	-2.26995	1.29075
Cl	1.32607	0.88245	-1.87078
Cl	-0.15798	-2.10016	-2.18499
Cl	-1.74243	0.36113	-0.46771
Cl	1.06442	0.67837	1.54969
H	0.67132	2.40523	-0.63499
H	0.39423	3.41803	0.68188
H	-0.70104	2.24496	0.22453
C	-2.97829	5.90295	1.43403
H	-3.38935	6.70453	0.80749
C	-1.73961	5.27426	0.81354
H	-1.28075	4.61127	1.56528
H	-0.99377	6.05908	0.59228
H	-0.95927	3.30302	-1.95331
N	-0.05081	2.97570	-0.12471
H	-3.76881	5.15288	1.58005
C	-2.01872	4.48583	-0.46790
C	-2.68747	5.34171	-1.53686
H	-2.08799	6.24102	-1.74784
H	-3.68510	5.67198	-1.22566
C	-0.74522	3.90026	-1.05738
H	-0.03392	4.69022	-1.33784
H	-2.68996	3.64242	-0.22022
H	-2.75089	6.33872	2.41423
H	-2.15301	-2.45804	-1.47382
H	-2.14216	-2.66214	0.14059
H	-2.75443	-3.86999	-0.83210
H	-3.37603	-0.76851	1.71521
H	-4.99137	-0.79457	2.46829
C	-4.91502	-2.91008	0.51586
H	-4.99439	-3.99230	0.29897
C	-6.32399	-2.32796	0.48487
H	-6.30838	-1.23445	0.59152
H	-6.93139	-2.73305	1.30464
C	-4.10651	-2.27593	-0.60616
H	-3.96758	-1.19779	-0.45689
N	-2.74046	-2.85423	-0.70440
C	-4.04259	-1.34109	2.37600
H	-3.56554	-1.34370	3.36336
C	-4.25468	-2.76957	1.89378
H	-3.28650	-3.29711	1.90391
H	-4.59758	-2.42172	-1.57689
H	-4.88538	-3.31322	2.61474
O	2.27656	-1.66348	0.00533
C	3.42829	-1.71323	-0.79152
C	4.69092	-1.59805	0.00459
C	4.86486	-0.26347	0.74333
C	6.15883	-0.31236	1.55634
C	6.39588	0.91526	2.42225
N	3.38221	-1.88281	-2.03405
C	4.83269	0.90194	-0.23589
H	6.57573	1.81406	1.81805
H	5.52730	1.11652	3.06629
H	7.26889	0.78013	3.07348
H	4.73352	-2.43031	0.72786
H	5.51886	-1.74010	-0.70377
H	4.03165	-0.12595	1.46081
H	3.90572	0.91327	-0.82569
H	5.67671	0.83388	-0.94082
H	4.89923	1.86548	0.28601
H	7.00652	-0.45028	0.86221
H	6.14174	-1.21013	2.19624
H	-6.83594	-2.56047	-0.45881
H	-2.80424	4.78741	-2.47745
H	2.48359	-1.39427	0.92237
H	2.41973	-1.92744	-2.38542

[$RhCl_5(L^4)$]: N-bonded-amide-3

Rh	-1.16934	-0.14771	0.00564
Cl	-0.88212	0.69999	-2.22906
Cl	-1.24174	-0.87838	2.34429
Cl	-2.64414	1.68454	0.53507
Cl	-2.94192	-1.54076	-0.65817
Cl	0.47294	-1.90802	-0.43558
N	0.43059	1.17568	0.84486
C	1.33414	1.85684	0.00971
C	2.31886	0.99104	-0.72554
C	3.20519	0.11658	0.16375
C	4.06013	-0.79095	-0.71886
C	4.79342	-1.87989	0.05014
O	1.28763	3.06849	-0.10318
C	4.04901	0.96696	1.10756
H	5.52056	-1.46970	0.76751
H	4.07151	-2.49101	0.61147
H	5.34222	-2.55086	-0.62665
H	1.72787	0.34625	-1.39692
H	2.93963	1.66558	-1.33614
H	2.55016	-0.55443	0.74597
H	3.42333	1.61912	1.73487
H	4.72630	1.62208	0.53320
H	4.66237	0.35390	1.78405
H	4.77837	-0.17084	-1.28880
H	3.39103	-1.26527	-1.45259
H	0.80093	0.41462	1.41757
H	-0.16484	1.81089	1.38179

[$RhCl_5(L^4)$]: N-bonded-enol-2

Rh	1.28250	-0.14063	0.00592
Cl	2.23369	-0.04192	2.23124
Cl	0.13003	-0.18241	-2.14948
Cl	1.77605	2.24388	-0.32766
Cl	3.37454	-0.75456	-0.98228
Cl	0.63484	-2.43949	0.39092
N	-0.56185	0.25566	0.85162
C	-1.44298	1.16615	0.68097
C	-2.88405	0.96727	1.06483
C	-3.58590	-0.03269	0.13596
C	-5.01694	-0.25784	0.62480
C	-5.77074	-1.34850	-0.12267
O	-1.19100	2.32775	0.10555
C	-3.52321	0.44045	-1.31031
H	-5.94672	-1.07614	-1.17203
H	-5.19811	-2.28746	-0.12087
H	-6.75005	-1.55008	0.33566
H	-2.93500	0.60386	2.10353
H	-3.40235	1.93881	1.02278
H	-3.03997	-0.99279	0.19990
H	-2.48070	0.52432	-1.65389
H	-4.00756	1.42682	-1.41423
H	-4.02427	-0.26463	-1.98801
H	-5.57159	0.69643	0.55101
H	-4.98914	-0.51109	1.69886
H	-0.88695	-0.60092	1.30238
H	-0.21170	2.36705	-0.14528

[$RhCl_5(L^4)$]: O-bonded-amide-1

Rh	1.19261	-0.17514	0.03412
Cl	1.90058	-0.92890	2.19790
Cl	0.37078	0.66282	-2.13655
Cl	2.32281	1.97681	0.39534
Cl	3.09319	-1.07736	-0.98928
Cl	-0.06356	-2.20540	-0.29789
O	-0.68084	0.54698	0.98266
C	-1.33921	1.50639	0.57909
C	-2.85063	1.41505	0.63741
C	-3.36258	0.06127	0.14784
C	-4.81314	-0.13612	0.58586
C	-5.39337	-1.49176	0.20820
N	-0.79541	2.67264	0.16564
C	-3.18483	-0.05920	-1.35976
H	-5.49790	-1.59766	-0.88016
H	-4.73315	-2.30180	0.55066
H	-6.38653	-1.64815	0.65538
H	0.22934	2.63432	0.02935
H	-1.30872	3.19044	-0.53643
H	-3.13351	1.56723	1.69296
H	-3.31654	2.23649	0.06286
H	-2.73182	-0.71111	0.62190
H	-2.13921	0.12299	-1.65582
H	-3.83648	0.66449	-1.88508
H	-3.42930	-1.06882	-1.71648
H	-5.43694	0.66900	0.14995
H	-4.87420	-0.00966	1.68102

[$RhCl_5(L^4)$]: O-bonded-enol-3

Rh	-1.63495	0.27302	0.22799
Cl	-3.49570	-0.92030	1.08687
Cl	0.21461	1.71224	-0.43956
Cl	-0.19691	-0.87103	1.78858
Cl	-1.28992	-1.20521	-1.49186
Cl	-3.11098	1.64573	-1.05414
O	3.01050	1.97812	0.97503
C	3.94391	1.42526	0.19919
C	3.45268	0.43379	-0.82424
C	2.84246	-0.83247	-0.21069
C	2.36672	-1.75096	-1.33465
C	1.70571	-3.03198	-0.84777
C	3.82236	-1.51767	0.73129
H	2.42617	-3.71260	-0.36701
H	0.91048	-2.79418	-0.12575
H	1.23659	-3.57306	-1.68191
H	2.68363	0.91339	-1.45168
H	4.29825	0.15399	-1.47779
H	1.94721	-0.54716	0.36721
H	4.17655	-0.83071	1.51307
H	4.70743	-1.88840	0.18033
H	3.35060	-2.37116	1.23680
H	3.22602	-1.98901	-1.99558
H	1.63488	-1.19129	-1.93970
H	2.09257	1.75652	0.66834
H	5.74814	1.21622	-0.24331
N	5.16974	1.74948	0.40714
